# Exploring the Versatility and Sustainability of Hydroxypropyl Methylcellulose (HPMC) in Modern Chemical Industry

**DOI:** 10.3390/polym18091105

**Published:** 2026-04-30

**Authors:** Sonia Matilla, Ackmez Mudhoo, Carlos Díez, Marta Otero

**Affiliations:** 1 Departmento de Química y Física Aplicadas, Universidad de León, Campus de Vegazana, 24071 León, Spain; smatid00@estudiantes.unileon.es; 2 Laboratorios León Farma, S.A., Insud Farma, C/La Vallina, Navatejera, 24193 León, Spain; carlos.diez@chemogroup.com; 3 Department of Chemical and Environmental Engineering, Faculty of Engineering, University of Mauritius, Réduit 80837, Mauritius; a.mudhoo@uom.ac.mu

**Keywords:** cellulose derivatives, thermogelable polymers, biodegradable polymers, sustainability, green chemistry

## Abstract

Hydroxypropyl methylcellulose (HPMC) is a cellulose derivative characterized by physicochemical properties (gel formation, water solubility, biodegradability, and biocompatibility). These properties explain their wide use in industries such as pharmaceuticals, food, and construction. This review evaluates the classification, production processes, and analytical characterization of HPMC, with particular attention to its versatility and sustainability life cycle. The environmental impact of HPMC is analyzed through its energy-intensive production, waste generation, emissions, and end-of-life biodegradability. In comparison with many petroleum-based polymers, HPMC is often considered a greener option and its use as an additive in modern chemical industry is extended. Therefore, the adoption of more sustainable production practices is essential to minimize its ecological footprint. In this sense, greener raw material sourcing, improved production process efficiency, lower emission etherification and purification routes, and broader implementation of life-cycle-based optimization strategies were identified as key priorities to be addressed.

## 1. Introduction

Hydroxypropyl methylcellulose (HPMC), also known as hypromellose, is a semi-synthetic cellulose ether obtained through the chemical modification of natural cellulose, in which hydroxyl groups are partially substituted with methyl and hydroxypropyl groups. This modification provides HPMC with unique physicochemical properties such as water solubility, gel-forming ability, film-forming capacity, versatile rheological behavior, and biocompatibility. As a result, HPMC is widely used in the pharmaceutical, food, cosmetic, and construction industries. Its non-ionic nature, broad stability over a wide pH range, and low toxicity have facilitated its common use in controlled-release systems, coating, and solidifying agents, and as a structuring agent in various formulations [1].

For example, in the pharmaceutical field, HPMC is an essential excipient for the design of innovative dosage forms, especially in controlled and modified release formulations, acting as a binder, matrix former, and film-forming agent [2]. HPMC is available in a wide range of grades. HPMC is commercially available in a wide range of grades that differ mainly in methoxy and hydroxypropyl substitution levels, molecular weight (MW), viscosity, particle size, and purity specifications depending on the intended use. These differences strongly influence key functional properties such as solubility, hydration rate, thermal gelation, water retention, film formation, and drug-release behavior, which explains why different HPMC grades are selected for different industrial applications [3]. However, the benefits of HPMC are achieved only when its stability and shelf life are controlled and evaluated properly [4]. HPMC stability and performance can change substantially depending on moisture levels, storage conditions, packaging, and compatibility with other excipients, which can impact on the efficacy, quality, and safety of the finished product. In a broader perspective, recent advances point to the growing use of biomass-derived materials and show that renewable polymers may be developed not only as sustainable alternatives to fossil-based materials, but also as platforms for high-value functional applications. Within this context, HPMC is particularly relevant because it combines the intrinsic advantages of cellulose-derived materials with tunable substitution patterns, viscosity behavior, film-forming capacity, and a wide applicability in pharmaceutical, food, and construction sectors [5,6].

Regarding all applications, interest in industrial sustainability has increased the need to evaluate the environmental footprint of HPMC over its life cycle. HPMC has several advantages compared with synthetic polymers, since it is cellulose-based, biodegradable, and non-toxic. However, HPMC production is still energy-intensive and may generate emissions and waste that require careful management [7,8,9,10]. Therefore, further progress in green manufacturing, emission reduction, and broader life cycle assessment remains necessary [9,11,12,13].

In the above-described context, the present review manuscript covers the following topics:

- HPMC characterization by reported analytical protocols and its classification.
- Raw materials and manufacturing.
- HPMC primarily in food, construction, and pharmaceuticals.
- Environmental impacts.
- Life cycle assessments considerations.
- Concluding thoughts and future directions.

The manuscript is organized to follow the progression from HPMC structure and physicochemical characterization, to classification, manufacturing, application, and finally environmental impact and life cycle considerations. This sequence is intended to highlight the relationship between molecular design, process conditions, functional performance, and sustainability.

This article places greater emphasis on the recent literature published between 2020 and 2026 in order to provide an updated and multidisciplinary overview of HPMC as a versatile and sustainable material. The literature survey was conducted using major scientific databases, including Google Scholar, PubMed, Web of Science, and Scopus. Scientific articles, review papers, industry reports, and international regulations were considered. Earlier references were retained only in limited cases when they were considered essential for the foundational understanding of HPMC’s structure, its characterization and its functionality. This review aims to highlight both the challenges and opportunities for optimizing the industrial production and use of HPMC in compliance with quality and environmental sustainability standards.

## 2. Physicochemical Properties and Analytical Techniques for Hydroxypropyl Methylcellulose Characterization

### 2.1. Physicochemical Properties

**Figure 1:**
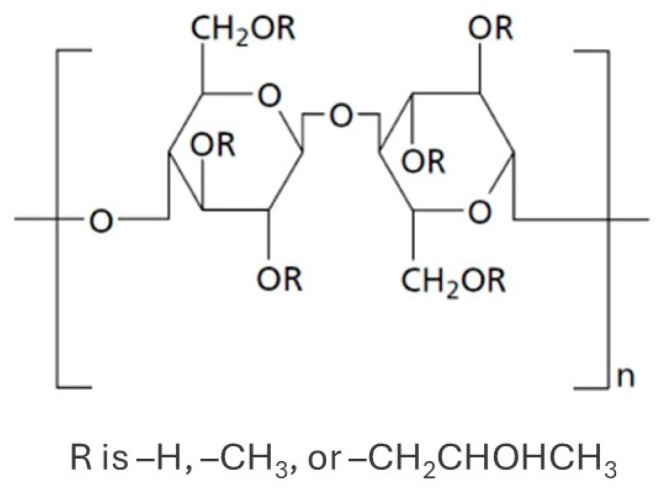
Chemical structure of HPMC, where some of the hydroxyl groups (–OH) in the anhydro glucose units of cellulose are substituted by hydroxypropyl (–CH_2_CHOHCH_3_) and methoxy (–OCH_3_) groups (adapted from Amanzholkyzy et al. [15]).

HPMC is a semi-synthetic, non-crystalline (amorphous) polymer derived from cellulose (polymer chains of β-ᴅ-glucose units connected by a beta acetal linkage) by its chemical modification [1]. It belongs to the group of cellulose ethers in which one or more of the three hydroxyl groups (–OH) present in the anhydro glucose unit of cellulose have been substituted by methoxy (–OCH_3_) and hydroxypropyl (–CH_2_CH(OH)CH_3_) groups, as shown in Figure 1.

The empirical formula of HPMC is C_8_H_15_O_8_-(C_10_H_18_O_6_)n-C_8_H_15_O_8_, which reflects a linear polysaccharide cellulose chain with ether-linked methoxy and hydroxypropyl side groups. Since these substituents are neutral, non-acidic, and non-basic, HPMC is a non-ionic electrically neutral polymer with electrolyte compatibility, low reactivity, and stability across a wide pH range (usually 3–11) [1]. These improved properties compared to natural cellulose are highly interesting for its diverse applications in modern industry.

Regarding the appearance of HPMC, it is a white/creamy white fibrous or granulated powder that is odorless, tasteless, and nontoxic [14]. In addition, the non-ionic nature of HPMC ensures consistent drug release profiles, as it is less affected by changes in pH and ionic strength of the dissolution medium. Further details on the main physicochemical properties of HPMC are described.

#### 2.1.1. Substitution and Viscosity Types

There are two substituents in HPMC, namely the methoxy and hydroxypropyl groups. Accordingly, the substitution pattern is respectively described by: (i) the methoxy degree of substitution (DSMe), which is the average number of hydroxyl groups substituted by methoxy groups in each cellulose unit (since each unit contains three hydroxyl groups, the maximum theoretical DSMe is 3); and (ii) the hydroxypropyl molar substitution (MSHP), which is the average number of hydroxypropyl groups in each cellulose unit (unlike DSMe, there is not a maximum MSHP, since hydroxypropyl substituents also contain hydroxyl groups that may be further substituted). Commercially available HPMC grades typically have DSMe ≈ 1.3–2.1 and MSHP ≈ 0.1–1.0 [3].

Viscosity reflects how much fluid resists flowing, which is especially important for HPMC when it is moved through pressurized piping systems. As a result, the grade in which HPMC is commercialized reflects its viscosity, namely that of a 2% aqueous solution at a specific temperature (usually 20–25 °C). HPMC’s viscosity strongly depends on its molecular weight (MW), with a higher MW resulting in it becoming more viscous [2]. The viscosity of HPMC also depends on DSMe and MSHP in a nonlinear complex correlation. HPMC is available in various viscosity grades, ranging from 3 to 100,000 cP, and the specific HPMC viscosity must be taken into account when engineering the target application. For example, in view of its use for drug release, high-viscosity HPMC is suitable for very soluble drugs, while low-viscosity grades are used for poorly soluble drugs [16].

#### 2.1.2. Thermal Gelation

Thermal gelation, which is the transition of an aqueous HPMC solution from liquid to gel when heated, is thermo-reversible in the case of HPMC (i.e., the gel returns to the liquid state when cooled). This is due to the amphiphilic non-ionic nature of HPMC, with hydrophilic regions (hydroxypropyl and hydroxyl groups) and hydrophobic regions (methoxy groups). At relatively low temperatures, polymeric chains are hydrated (hydrogen bonds with hydrophilic regions) and HPMC is fully dissolved. However, as temperature increases, the hydrogen bonds break, methoxy groups aggregate and polymeric chains associate, leading to the HPMC liquid solution becoming a gel (3D network) at the gelation temperature. This gelation specifically occasions the subsequent viscosity increase. The higher DSMe and the lower MSHP result in a lower gelation temperature of HPMC, which in turn also decreases with decreasing MW. In general, the hydration process of HPMC’s polymeric structure is thermally dependent, with sol–gel transitions happening at relatively high temperatures (typically 60–80 °C) [17]. In order to decrease the gelation temperature, and when this is favorable for the target application, salts and additives are usually incorporated into HPMC structure besides turning DSMe and MSHP. This may be interesting, for example, for ophthalmologic or controlled-release tablets, which need HPMC to gel in the eye or in the stomach at the body temperature. In fact, HPMC gelation is thermo-reversible. This reversibility is conditioned by its properties and concentration in the specific solvent, the presence of additives and their nature, and the thermal environment [18]. The substitution pattern (DSMe and MSHP) and the ratio of methoxy and hydroxypropyl groups not only heavily influence viscosity but also impact other physicochemical properties of HPMC, such as thermal gelation, solubility, swelling, powder flow, compressibility, compactability, and diffusion [19,20].

#### 2.1.3. Molecular Weight

HPMC is a heterogeneous, polydisperse polymer. Its MW corresponds to average polymer chain mass, which is usually estimated by viscosity or light scattering measurements. It is usually expressed in kilodaltons (kDa), with typical HPMC MWs ranging from 10 kDa to 1500 kDa [21]. One of the key factors contributing to the differences in MW is the polymeric length of HPMC chains. Polymers with longer chains generally possess higher MW. On the other hand, for the same chain length, MW increases with substitution due to the added mass of the methoxy and/or hydroxypropyl groups.

The MW of HPMC may be expressed by *M_n_* (number average MW) or *M_w_* (weight average MW), which are calculated as follows:

(1) $$M_{n}=\frac{\sum n_{i}M_{i}}{\sum n_{i}}$$

(2) $$M_{w}=\frac{\sum n_{i}{M_{i}}^{2}}{\sum n_{i}M_{i}}$$

where *n_i_* is the number of HPMC polymer chains and *M_i_* is the corresponding molecular mass. *M_n_* is a simple average while *M_w_* weighs each molecule by its mass contribution ${(M}_{w}\geq M_{n}$). In this way, long chains have priority and consequently result in HPMC viscosity. The ratio between these values is the polydispersity index $\left(PDI=\frac{M_{w}}{M_{n}}\right)$, which indicates how wide the MW distribution is and it usually takes values between 1.5 and 3.0 for HPMC [21].

The MW and polymer chain length of HPMC are key factors that affect its functional properties in a wide range of applications, including pharmaceutical formulations. In general, HPMC with a higher MW shows higher viscosity (≥10,000 mPa·s in a 2% aqueous solution at 20 °C) because it forms more extensive and stronger three-dimensional polymer networks [22]. These dense networks result in slower dissolution and remarkably affect controlled-release drug formulations, slowing down delivery whereby prolonged release is required. In contrast, lower MW HPMC (≤100 mPa·s at 20 °C and in 2% aqueous solution), hydrate and dissolve faster, causing quicker delivery. Thus, MW needs rigorous monitoring because it is a critical factor for the product performance in any specific application [23].

#### 2.1.4. Water Solubility

HPMC is soluble in water, where, upon hydration, and depending on its grade and concentration, it forms a clear to slightly turbid colloidal solution. HPMC exhibits higher solubility in cold water, this being because, at lower temperatures, hydrogen-bonding interactions between the hydrophilic groups of the polymer and water molecules promote hydration and chain dispersion. When the temperature rises, hydrophobic interactions among substituted polymer segments become more significant, in turn decreasing solubility and promoting intermolecular association that finally leads to thermal gelation. Consequently, HPMC does not follow the usual dissolution profile commonly observed with many small molecular compounds. In water, HPMC dissolves for a pH ranging 3–11, with solubility decreasing outside this range. On the other hand, HPMC is normally insoluble in organic solvents (e.g., chloroform and acetone), or only slightly soluble, often forming suspensions in lieu of true solutions. The solubility characteristics are influenced by MW of the polymer and the degree of substitution. High-quality HPMC dissolves fast and homogeneously in water without lump or gel formation [19].

The dissolution of HPMC in water involves the following steps: (i) hydration through the penetration and adhesion of water molecules to hydrophilic groups (mainly hydroxyl and hydroxypropyl groups) by hydrogen bonds; (ii) breaking down of interactions between polymeric chains due to adhesion of water molecules; and (iii) the gradual separation and dispersion of polymer chains in solution. Therefore, HPMC’s water solubility points to its ability to hydrate and disperse in water, for forming a stable homogeneous clear or slightly turbid viscous solution. Consequently, relatively high MSHP/DSMe ratios with uniform substitution patterns favor water solubility. Contrarily, high MW results in relatively lower HPMC water solubility. Low-viscosity grades of HPMC dissolve more easily in water while high-viscosity grades are more difficult to get hydrated and disperse in water at high concentrations. On the other hand, HPMC water solubility is higher at lower temperature and decreases with the increase in temperature, which causes HPMC chain aggregation with formation of gels (thermal gelation), as referred above [24].

It is necessary to highlight that cellulose is not soluble in water but, due to substitution, HPMC is mostly amorphous, allowing water to penetrate its structure, form hydrogen bonds with hydroxyl and hydroxypropyl groups and solvate and disperse the polymer chains. The water solubility of HPMC is a significant advantage over cellulose, enabling its use in different applications. Since its aqueous solutions are odorless and tasteless, HPMC may be used for formulations in pharmaceutical, food, and cosmetics industries. Furthermore, since its structure does not contain ionizable groups or permanent charges, aqueous solubility of HPMC is not remarkably affected by pH (within 3–11) or ionic strength [2]. This is also a pertinent attribute of HPMC because body fluids contain electrolytes and proteins/enzymes. These characteristics make possible HPMC’s applications in the following: in pharmaceutical controlled-release matrices and film coatings; as a thickener, stabilizer, or emulsifier in the food industry; or to provide viscosity to different cosmetics or personal care products (lotions, creams, shampoos, etc.) [25,26].

High water solubility allows for easier preparation of HPMC solutions and is beneficial for quick solutions, coatings, and applications in spray, while HPMC with relatively lower solubility in water is more adequate for stable gels or applications requiring long-term release. Therefore, it is essential that HPMC possesses adequate and consistent properties that make it appropriate for the target application. Furthermore, temperature and mixing parameters need to be adjusted to optimize HPMC’s solubility and performance [27].

Although HPMC is soluble, its non-ionic nature makes it useful for multiple applications, for example, it reduces the risk of drug interactions and usually provides reproducible drug release profiles, as gastrointestinal fluid pH does not significantly affect it [22].

In general, HPMC’s chemical structure and physicochemical properties make it an essential component for industrial applications, with an especially relevant role in the pharmaceutical industry, offering solutions for drug solubility and controlled release challenges [28].

### 2.2. Analytical Techniques and Methodologies for Hydroxypropyl Methylcellulose Characterization

Section 2.2 focuses on the analytical identification and characterization of HPMC, emphasizing how each technique contributes to confirming its chemical substitution, structural features, molecular properties, and grade-related functional behavior. The analytical characterization of HPMC is essential for ensuring quality and consistency (e.g., MW, DSMe, MSHP, viscosity, etc.), determining functional properties (since these depend on physicochemical properties), checking standards or regulatory compliances [1]. Therefore, analytical characterization of HPMC is necessary in view of its industrial application, especially for the pharmaceutical, food, cosmetics, and construction industries, and for research in polymer science, as well as in materials and products development [29,30].

Different analytical techniques have been used for the characterization of HPMC [31,32]. For the chemical composition of HPMC, Nuclear Magnetic Resonance (NMR (^1^H, ^13^C)) spectroscopy is used to determine DSMe, MSHP, and substitution pattern while Fourier Transform Infrared Spectroscopy (FTIR) allows for a qualitative identification of functional groups. For further evaluation of the functional groups, Raman spectroscopy is commonly used. Also, purity and/or impurities are usually assessed by high-performance liquid chromatography (HPLC) and gas chromatography coupled to mass spectrometry (GC-MS). As for MW, it is usually assessed by Gel Permeation Chromatography/Size Exclusion Chromatography (GPC/SEC), which allows for measuring *M_n_*, *M_w_*, and *PDI* (polydispersity index; defined as *M_w_/M_n_*, i.e., the ratio of weight-average to number-average molar mass) together with capillary viscometry, which allows for intrinsic viscosity determination and MW estimations. Physical characterization is usually carried out by viscosity testing in order to determine HPMC grade and by Thermal Analysis, namely Differential Scanning Calorimetry (DSC) and Thermogravimetric Analysis (TGA), so to assess thermal gelation, degradation, and/or moisture content. Crystallinity vs. amorphous state of HPMC can be determined by X-ray diffraction (XRD), which also allows the assessment of homogeneity and stability. Regarding particle size distribution, it is usually determined by laser diffraction, while particle shape and surface morphology are assessed by Transmission Electron Microscopy (TEM) and Scanning Electron Microscopy (SEM). A brief description of the most commonly used analytical techniques for HPMC characterization is presented in Table S1 within Supplementary Materials depicting a summary on their use [33,34].

#### 2.2.1. Scanning Electron Microscopy (SEM)

SEM is a multifunctional instrumental technique that images and analyzes bulk specimens by detecting interactions between irradiated electrons and the sample surface. It provides high-magnification, high-resolution images of surface texture and structure using secondary and backscattered electrons [35].

SEM is primarily used to study the size, shape, and surface morphology of particles. This is especially useful for HPMC, which is commercialized as a powder, since particle characteristics strongly influence its behavior and performance in formulations. SEM allows detailed visualization of particle homogeneity, surface texture, and structural features, making it a key technique for the physical characterization of HPMC in various industrial and research contexts [34].

For instance, SEM has been used to show that HPMC helps maintain the texture and quality of nutrition bars by preventing hardening during storage through modification of sugar crystallization, thus contributing to a softer texture and longer shelf life [36]. This example illustrates the use of SEM for industrial quality control of HPMC, although complementary characterization by other analytical techniques is usually necessary.

#### 2.2.2. Transmission Electron Microscopy (TEM)

TEM produces images by passing electrons through a thin sample, allowing the internal structure to be seen in very high detail [35]. TEM is used to examine the internal morphology and fine structural features of HPMC particles and films. It helps reveal sub-particle characteristics, particle dispersions, and nanoscale interactions, which are important for understanding and improving the behavior of HPMC in formulations. TEM can also show how HPMC interacts with excipients and how processing affects its microstructure, which has an impact on properties such as dispersibility, film formation, and mechanical strength [37].

For example, TEM was used in a study exploring spray drying co-processing of HPMC with lactose and sodium chloride to enhance dispersibility. The resulting TEM images showed changes in the mixing and internal structure of co-processed HPMC particles, which correlated with improved dissolution times and film properties, demonstrating that the technique can support formulation development and excipient selection [29].

#### 2.2.3. Fourier Transform Infrared Spectroscopy (FTIR)

FTIR spectroscopy is based on the vibration and rotation of atoms and has become a universal and widely used spectral method across many fields [38]. In the case of HPMC, FTIR spectroscopy provides confirmation of the characteristic functional groups, such as hydroxyl, methyl, and ether groups, and allows qualitative inference of the degree of substitution [32]. HPMC exhibits specific FTIR signals that correspond to these groups. The methyl group (–CH_3_) in methoxy (–OCH_3_) substitutions contributes to C–H stretching vibrations around 2840–2950 cm^−1^, while C–H bending vibrations near 1375–1450 cm^−1^, and C–O (ether linkage) stretching between 1050 and 1150 cm^−1^, allow confirmation of a presence of methoxy groups attached to the cellulose backbone. Additionally, hydroxypropyl substitution (–CH_2_CH(OH)CH_3_) also contributes to C–H stretching in the 2840–2950 cm^−1^ region due to extra methyl and methylene groups, overlapping C–O stretching in the 1000–1100 cm^−1^ region, and a broad O–H stretching band (~3500–3400 cm^−1^) arising from hydroxyl (-OH) groups [38,39].

The band near 895 cm^−1^, characteristic of native cellulose and indicative of the β (1→4) glycosidic bond, generally remains present in HPMC, although it may be slightly shifted or reduce intensity. The broad O–H band (~3400 cm^−1^) typical of cellulose is often narrowed or reduced in HPMC due to substitution, indicating a lower number of free –OH groups. Furthermore, the C–O and C–O–C bands (~1050–1150 cm^−1^) become more complex in HPMC because of the additional ether bond introduced by methoxy and hydroxypropyl groups [40,41].

FTIR characterization of HPMC is crucial in both the pharmaceutical industry and research to ensure the quality and consistency of the polymer. For instance, it allows verification of the degree of substitution and chemical integrity, which are two critical parameters for HPMC’s performance as an excipient in controlled-release drug formulations [42]. Moreover, FTIR helps detect impurities or chemical modifications that could affect the functionality of the final product, consequently fully supporting quality control and the development of new HPMC-based materials [43].

#### 2.2.4. Raman Spectroscopy

Raman spectroscopy is based on the Raman effect, in which a small fraction of the scattered light differs in frequency from the incident monochromatic radiation. This technique relies on the inelastic scattering of light arising from its interaction with molecular vibrations. In the characterization of HPMC, Raman spectroscopy provides detailed information about molecular vibrations and chemical structure without requiring extensive sample preparation. Raman spectroscopy can be used to identify functional groups in HPMC, such as hydroxyl, methyl, and ether groups. Changes in band position and intensity can also provide information on the degree of substitution and molecular conformation. The technique helps to differentiate various substitution patterns along the cellulose backbone. It also aids in the assessment of the homogeneity and purity of HPMC samples. It is a valuable technique for examining molecular interactions and structural changes in HPMC under various processing and environmental conditions because it is non-destructive and chemically specific. HPMC’s Raman spectrum reveals important information on its chemical groups and backbone structure. For instance, FTIR bands at 1384.3 cm^−1^ and 908.3 cm^−1^ are characteristic of HPMC. The 1384.3 cm^−1^ band corresponds to vibrations of methyl/methylene groups in the methoxy and hydroxypropyl substituents, and the band near 908.3 cm^−1^ is associated with the pyranose ring of the cellulose backbone, thus validating the integrity of the polysaccharide ring system. These specific Raman shifts enable identification and monitoring of HPMC’s degree of substitution and molecular conformation. The sensitivity of Raman spectroscopy to these molecular vibrations makes it very effective for non-destructively assessing the substitution patterns, chemical composition, and structural features of HPMC [42].

Raman spectroscopy is extensively employed both in industry and research for quality control and in the design of HPMC formulations. For instance, the chemical composition and extent of substitution of HPMC batches can be crosschecked to ascertain consistency in pharmaceutical excipients [22]. In research, this technique aids in elucidating HPMC’s behavior at the molecular level in drug delivery systems. Such information can then be harnessed to enhance HPMC’s performance and stability. Raman spectroscopy is also employed to monitor production processes and to develop new HPMC-based materials [44].

#### 2.2.5. X-Ray Diffraction (XRD)

XRD is used to study how X-rays are diffracted by the structure of a material. For HPMC, it is mostly employed for assessing the degree of crystallinity and structural parameters. HPMC being mostly amorphous, its XRD pattern usually comprises broad halos in lieu of sharp peaks, thus depicting its disorderly molecular structure. HPMC’s amorphous character affects its properties (viz. stability, solubility, and mechanical behavior), that collectively can determine its performance in different applications. XRD can also assist in detecting residual crystallinity or changes in structural order resulting from chemical modification or processing, thus making it a valuable complement to microscopic and spectroscopic analyses. Because XRD examines the sample in its entirety, it equally assists in confirming if features depicted in a small number of particles under the microscope are (adequately) representative of the bulk material [45].

In pharmaceutical applications, XRD elucidation on HPMC being amorphous or with a certain crystallinity degree helps in the assessment of controlled-release drug delivery formulations. In research, XRD assessment on how processing or chemical modification affect HPMC’s structure is valuable for designing tailor-made materials for use in coatings, drug delivery, and food applications [46].

#### 2.2.6. Nuclear Magnetic Resonance (NMR)

In HPMC characterization, NMR spectroscopy is extensively employed in studying its molecular structure and substitution patterns. Proton NMR (^1^H NMR) is useful to estimate the amount of hydroxypropyl groups and glucose units in the polymer, thus providing data on composition and degree of substitution, while Carbon NMR (^13^C NMR) allows elucidating how hydroxypropyl and methoxy groups are distributed along the cellulose backbone. Together, they help explain how chemical modifications in HPMC are linked to its physical and functional characteristics [47]. This is particularly relevant for pharmaceutical applications, namely, to determine how HPMC behaves as an excipient in drug delivery formulations. In research, NMR is useful for quality control and development of novel HPMC derivatives with specific properties, since it gives thorough information about molecular structure and behavior.

#### 2.2.7. Differential Scanning Calorimetry (DSC)

DSC, which measures heat absorption or release with temperature, is useful in determining materials thermal properties and phase transitions [48], being commonly employed for HPMC characterization. Unlike crystalline polymers, HPMC does not usually have a clear melting peak. Rather, its glass transition temperature (Tg), typically ~170–180 °C, indicates the transition from a rigid to a more flexible state. DSC is also useful in determining the onset of thermal degradation. Also, DSC analysis helps to detect moisture loss by endothermic evaporation. Since substitution type and MW can affect both Tg and degradation onset, DSC is particularly relevant for comparing different HPMC grades and their respective thermal characteristics. DSC can also assist in evaluating polymer compatibility and miscibility. For instance, if the melting peak of a drug disappears when mixed with HPMC, this may point to the formation of an amorphous dispersion, often linked to improved bioavailability [49].

In pharmaceutical development, DSC is useful for optimizing processes such as coating, drying and controlled release. In research, DSC is relevant in the development of novel HPMC mixtures having specific mechanical and thermal features, supporting both quality control and development of novel HPMC-based materials.

#### 2.2.8. Thermogravimetric Analysis (TGA)

TGA measures how the mass of a material varies when the temperature increases. Such information reveals the thermal stability and composition of the material under analysis. TGA is extensively used to study HPMC’s thermal degradation behavior. TGA can reveal the temperature at which decomposition starts (usually ~200–250 °C), and this explains the polymer’s thermal stability. Also, TGA can point to chemical interactions (e.g., possible esterification between HPMC and drugs like ofloxacin), which may impact performance in drug delivery systems, and give indirect information about hydrogen bonding in HPMC, which influence its solubility and behavior with other compounds. By analyzing degradation steps and mass loss profiles, TGA helps in understanding HPMC’s composition, stability, and thermal behavior, which is essential for the structural integrity monitoring required in controlled-release pharmaceutical applications [17].

Also, TGA characterization is crucial for quality control and formulation development. For example, understanding the thermal degradation profile ensures that HPMC-based drug delivery systems maintain stability during processing and storage. It also aids in optimizing formulations by monitoring chemical interactions with active pharmaceutical ingredients, to enhance drug release profiles. Moreover, TGA helps in the development of new HPMC grades with tailored thermal and mechanical properties, which is highly useful for specific applications, for example to support innovation in pharmaceutical excipients and controlled release technologies [17].

#### 2.2.9. Rheological Techniques

Rheological characterization is fundamental for understanding the flow and gelation behavior of HPMC. Two main analytical instruments are used for this purpose, namely capillary viscometers and rheometers [50]. A capillary viscometer is used to measure the viscosity of HPMC solutions under steady shear conditions, giving information about MW and interaction between polymer and solvent. Meanwhile, a rheometer allows for determining both shear viscosity and dynamic properties (e.g., loss modulus (G″) and storage modulus (G′)) at various shear rates and temperatures. Together, dynamic moduli and shear viscosity measurements explain the gelation process and the viscoelastic characteristics of HPMC [50].

Rheological studies of HPMC solutions, often at 2% *w*/*w*, are usually carried out to determine thermogelation properties, such as aggregation temperature (Tagr) and gelation temperature (Tgel) [51], which depend on the type and degree of substitution (e.g., methoxy content). As for MW, it generally has small influence on HPMC gelation temperature despite it affects viscosity. Rheological measurements are used in the study of HPMC–surfactant interactions and provide information on how strongly the polymer chains interact. This aspect is normally described by the overlap parameter (c/c*). This parameter compares the concentration of the polymer solution (c) with the concentration at which the polymer coils begin to overlap (c*).

In pharmaceutical formulation development and quality control, understanding HPMC’s thermogelation patterns helps optimize drug release in controlled-release systems. Measurement of viscoelastic properties and gelation temperature also help ascertain a consistent performance during manufacturing and storage. Additionally, rheological analysis guides the design of HPMC-based nanocomposites by evaluating filler dispersion and polymer-filler interactions, which affect mechanical and barrier properties. In research, rheology helps in the development of HPMC derivatives with specific substitution patterns to achieve the target of solubility, stability, and gelation behavior, supporting advances in excipient design and drug delivery systems [52].

The aforementioned physicochemical and analytical parameters enable an understanding of HPMC’s functional behavior. These attributes are both important in characterization and are the major criteria for HPMC’s classification by grades with different performance profiles. The next section explains how these structure and property-related attributes translate into the practical classification systems for industrial and formulation use.

## 3. Classification of Hydroxypropyl Methylcellulose

To better understand and use HPMC for specific applications, it is helpful to classify it according to the physicochemical properties that affect its behavior and performance. The main classification criteria include the degree of substitution of its functional groups, viscosity, and MW. Each of these factors determines solubility, gelation behavior, mechanical strength, and the release profile of active substances in HPMC-based formulations [53]. At this point, HPMC is widely used for the controlled release of many substances, including not just drugs but also nutrients, such as vitamins and minerals, as well as aromas, flavors, fragrances, and agrochemicals. The following sections give a more detailed overview of these classification methods and explain why they are important for selecting the most suitable type of HPMC for a given application [53].

### 3.1. Classification Based on the Substitution Degree

HPMC is classified based on the degree of substitution of its functional groups, specifically the relative amounts of methoxy and hydroxypropyl groups attached to the cellulose backbone. According to the United States Pharmacopeia (USP), there are four main types of HPMC—HPMC 1828, HPMC 2208, HPMC 2906, and HPMC 2910—where numerical codes indicate the approximate percentage of methoxy and hydroxypropyl groups in the polymer after drying at 105 °C for 2 h. For instance, HPMC 2208 consists of about 8% hydroxypropyl and 22% methoxy groups. The first two digits refer to the methoxy content, and the last two refer to the hydroxypropyl content. These differences in substitution especially affect hydrophilicity, thermal properties, and gel strength of HPMC.

Commercially, HPMC types are often identified by codes that reflect their substitution degree and viscosity. For instance, The Dow Chemical Company markets Methocel^®^ grades with designations such as K, E, and F, corresponding to USP types HPMC 2208 (K), HPMC 2910 (E), and HPMC 2906 (F), respectively. These codes are followed by suffixes indicating the viscosity of a 2% aqueous polymer solution at 20 °C, measured in centipoise (cps). The suffix “C” denotes viscosity multiplied by 100, while “M” denotes viscosity multiplied by 1000. Additional suffixes specify product grades, such as premium (P), low viscosity (LV), controlled release (CR), granular (G), direct compression (DC), surface treated (S), or food grade (FG) [54,55]. For example, METHOCEL™ J12MS corresponds to a HPMC grade of “J” chemistry with a nominal viscosity of 12,000 mPa·s (cP) in a 2% aqueous solution, and the suffix “S” indicates a surface-treated grade designed for easier dispersion in water [27].

The degree of substitution is a critical parameter that, together with viscosity, defines the functional characteristics of HPMC. For example, higher hydroxypropyl content generally increases hydrophilicity and gel strength, which is advantageous, for example, in sustained-release pharmaceutical formulations. HPMC is widely used as a hydrophilic matrix former in controlled-release tablets, and its substitution pattern can affect both drug release and tablet integrity [56,57].

As for classification and considering HPMC application as pharmaceutical excipient, recent research has mainly focused on four non-ionic methylated cellulose ethers: methylcellulose, HPMC E type (2910), HPMC F type (2906), and HPMC K type (2208), which mainly differ in their degree of substitution. These polymers are available in a wide range of MW and viscosity, giving formulation scientists flexibility when designing drug delivery systems. For example, 2% (w/v) aqueous solutions of HPMC K100 and HPMC K15M have nominal viscosities of 100 and 15,000 centipoise, respectively, showing the broad functional range that can be achieved by varying substitution and MW [56].

In summary, classification based on the degree of substitution is important for selecting the right HPMC grade for a given formulation, whether in pharmaceutical controlled-release systems, food applications, or industrial uses [1].

### 3.2. Classification Based on Viscosity

HPMC is available in many viscosity grades, mostly determined by its degree of substitution and MW. Low-viscosity grades generally have viscosities of a few centipoises, while high-viscosity grades are viscosities reaching 100,000 cP or higher. These grades are often identified by commercial codes such as E3, F4M or K100. A summary of these grades and their respective applications are given in Table 1. The gel-forming capability of high-viscosity HPMC supports its role as a matrix former in hydrophilic matrix tablets. Rheological measurements are regularly used to characterize the viscosity behavior of HPMC grades, with a view to ensuring that they embody a consistent quality and deliver high performance in formulation development [2]. Normally, MW more than 60,000 is regarded as high for HPMC. Values less than 50,000 are classified as ‘low’. These differences are much linked to HPMS’s viscosity. Grades having viscosities of 100–4000 cP are generally linked with faster release, while those with viscosities of 15,000–100,000 cP are usually applied in prolonged or controlled-release systems [22].

The classification of HPMC grades is not merely descriptive, it also reflects the outcome of specific production choices. Parameters such as methoxy/hydroxypropyl substitution, MW, viscosity, and particle size are ultimately established during synthesis and post-processing. For this reason, understanding HPMC classification naturally leads to consideration of the raw materials and manufacturing operations that determine these quality attributes. The next section therefore examines how production routes shape the final properties and performance of HPMC.

## 4. Raw Materials and Manufacturing Process

Once the key classification criteria of HPMC have been established, it becomes important to examine how these attributes are generated during production. The manufacturing process not only converts cellulose into a functional cellulose ether, but also determines the substitution pattern, viscosity range, purity, and particle properties that define each HPMC grade. Thus, production conditions are directly linked to the structure–property relationships described above and ultimately to the suitability of HPMC for specific industrial applications. Cellulose is the main raw material used to produce HPMC [11]. The process begins with cellulose purification to remove impurities and ascertain consistent final product quality. The purified cellulose is treated with NaOH to form alkali cellulose, an important intermediate in HPMC production. NaOH is extensively considered the most effective alkali for dissolving lignin and hemicellulose, and it is regularly used to enhance pretreatment efficiency in largescale industrial production [11]. The alkali cellulose is etherified (i.e., reaction with propylene oxide and methyl chloride), and this conversion introduces hydroxypropyl and methoxy groups within the cellulose structure, to finally yield producing HPMC. The degree of substitution and the ratio of these two groups can be adjusted to synthesize HPMC having tailor-made properties for various applications [58]. After etherification, the product is neutralized, washed to remove residual chemicals and by-products, and then dried. The dried HPMC is milled to obtain the desired particle size. Particle size is an important process variable because it influences dispersion behavior, hydration rate, gel layer formation, handling, and dust formation. Maintaining product quality and consistency demands a thorough understanding of the HPMC manufacturing process. This helps manufacturers enhance production efficiency and satisfy the requirements of different industries. HPMC production and post-processing comprise a series of defined steps ranging from raw material selection to final packaging, and each step should be thoroughly monitored to ensure a consistent product quality [59]. These steps are outlined below.

Reported information on HPMC production yield is limited in the open literature and depends strongly on the cellulose source, pretreatment efficiency, reaction conditions, etherification extent, and purification losses. In general, yield is influenced not only by the efficiency of substitution but also by side reactions, incomplete conversion, and material loss during washing and drying. In parallel, the cellulose degree of polymerization may decrease during alkaline activation and subsequent etherification because chain scission can occur under processing conditions. These changes are important because they affect the MW distribution and, consequently, the viscosity grade and performance of the final HPMC product [11].

### 4.1. Raw Material Selection

Cotton linters followed by wood pulp are the main industrial raw materials used for HPMC production because they provide relatively high cellulose purity, controlled composition, and process consistency. In contrast, many alternative cellulose sources may require more extensive pretreatment and purification due to higher contents of lignin, hemicellulose, ash, or other impurities, which can hinder reproducible etherification and product standardization. Cotton linters comprise a purer and more uniform source than wood pulp, which contains cellulose mixed with hemicellulose and lignin [58]. Cellulose purity is crucial in producing HPMC having a consistent quality. Impurities like hemicellulose, lignin, ash, or residual chemicals tend to lower reactivity during chemical modification and alter the final polymer’s physicochemical features. These impurities affect viscosity, solubility, and gelation capacity, which can in turn affect HPMC performance in food or pharmaceutical industrial applications. Therefore, effective purification and strict quality control are mandatory to have the best possible starting material [60].

Beyond cotton linters and wood pulp, alternative cellulose sources such as agricultural residues, energy crops, and bacterial nanocellulose have also been considered in the literature. Lignocellulosic biomass has been identified as a sustainable source of cellulose when adequate fractionation and purification are achieved, whereas bacterial nanocellulose is attractive because of its high purity, but it is still limited by cost, productivity, and scale-up constraints. Therefore, these sources are chemically feasible for HPMC production, but at present they should be regarded as emerging rather than established industrial feedstocks [61,62,63].

### 4.2. Etherification Reaction

The etherification reaction is critical in HPMC synthesis, and it requires precise control of many factors (viz. etherification agent, catalyst used, reaction conditions and the scope of process monitoring). The main etherification agents employed in HPMC production are propylene oxide and methyl chloride [64]. The type and ratio of these reagents have an important bearing on the final substitution pattern, and by ricochet, on HPMC’s functional behavior. Cellulose etherification is usually performed under strictly controlled reaction conditions involving pressure, temperature, and pH, for these parameters exert a direct impact on substitution pattern and reaction kinetics. In most production protocols, the process is carried out in an alkaline aqueous milieu. Reaction temperatures are usually maintained between 40 °C and 90 °C for a practical balance between enhancing etherification rate and minimizing thermal degradation or unwanted side reactions within the cellulose backbone [65]. Alkali catalysts such as NaOH or KOH have an essential role in the deprotonation of the hydroxyl groups along the cellulose backbone for producing the corresponding alkoxide species. These alkoxides are significantly more nucleophilic than the neutral hydroxyl groups, which enables them to interact more efficiently with the electrophilic centers of the etherification reagents. Thus, having a strong base both activates the cellulose and takes the overall reaction toward higher substitution efficiency [66]. Alkali catalyst type and concentration bear a pronounced effect on the reaction kinetics, the substituent distribution along the cellulose chain, and eventually the molecular-weight profile of the HPMC thus produced. However, strong bases can accelerate both etherification and unwanted side reactions. Thus, careful adjustment of catalyst concentration is crucial. Excess alkali may give rise to cellulose degradation through peeling or chain scission. Excessive harsh conditions can induce partial crosslinking. Therefore, optimizing the catalyst level is critical to ensure closely controlled substitution and for maintaining the polymer’s structural integrity. Thus, continuous monitoring and real-time control of the reaction conditions are vital to reach a consistent product quality and ensure reproducible results in a robust production system. This is because the process is highly sensitive to fluctuations in pH, temperature, or catalyst levels. Key parameters such as temperature, pH, pressure, and reaction time are tracked in real-time using automated sensors and control systems. Additionally, sampling during the reaction allows for off-line analysis of intermediate DSMe, MSHP and MW [65]. This monitoring provides adjustments of reaction conditions to maintain the target values of these parameters and avoid side reactions or degradation. Quality control at this stage ensures that the HPMC produced meets the stringent specifications required for its diverse applications.

### 4.3. Purification and Washing

After the etherification reaction, the resulting HPMC reaction mass contains the desired modified polymer together with unreacted cellulose, residual etherification agents (e.g., propylene oxide and methyl chloride), alkaline catalysts (NaOH or KOH), salts generated during downstream conditioning, and low-molecular-weight by-products [67]. These impurities can adversely affect the performance, safety, and stability of the final product; therefore, a purification process is required to ensure compliance with quality specifications [68].

Purification is typically based on a combination of (i) washing steps coupled to solid–liquid separation to remove readily extractable soluble residues (e.g., alkali and salts) and reduce the bulk of process contaminants, and (ii) more targeted impurity-removal measures as needed, such as pH adjustment/conditioning, precipitation, or coagulation, to separate insoluble fractions, as well as specific chemical treatments intended to degrade, transform, or eliminate the remaining unwanted substances.

Several washing cycles are needed to thoroughly cleanse HPMC. Washing is typically performed with plenty of water to remove leftover alkali, salts, and other water-soluble residues. Solvents such as ethanol or isopropanol may also be used if there are hydrophobic impurities or traces of etherification agents that poorly dissolve well in water. Washing should be performed so as to maximize impurity removal and minimize product loss. How many washing cycles are needed, the solvent type, the washing process temperature, and agitation regime must all be optimized for specific production scale and purity requirements. After washing, the HPMC is separated from the liquid by way of vacuum filtration, centrifugation, or pressure filtration. Which method to apply is determined by the viscosity, particle size, and volume of sample. After filtration, the wet HPMC is dried to drive out leftover solvent or moisture. Drying can be achieved by fluidized bed drying, tray drying or spray drying, depending on the product form and the polymer’s heat sensitivity requirements. The final dried material is generally a fine powder or granular, thus ready for milling, sieving, and packaging [69,70].

### 4.4. Particle Size Control

HPMC particle size control is important because it impacts HPMC’s dissolution, flow-related properties, compression, and performance in diverse applications [71]. A consistent particle size contributes towards reliable product quality. The main steps of such a control protocol are realized stepwise during the manufacturing process. These steps are now concisely revisited. Post drying, HPMC often tends to be clumped or take larger particles configurations. Thus, HPMC must be properly milled to the requisite particle size. This can be achieved by hammer milling, ball milling, pin milling, or jet milling. The choice of the specific milling regime depends on the material’s hardness, the final milled size, and HPMC’s heat sensitivity. Rotor speed, feed rate or throughput, and milling time are key variables requiring close control to ward off any overheating, that otherwise damages the HPMC or alters its sought properties [72]. The HPMC is sieved to sort the particles after the milling. This is done to reach a uniform particle size distribution. Sieving generates batches under strict particle-size control, which is an essential process component in pharmaceutical applications whereby dosing and consistency are essential [30]. There are many analytical methods commonly applied to measure particle size and distribution. Laser diffraction optical or electron microscopy, dynamic light scattering (DLS) can be used to this end. Such methods (used in combination when needed) help manufacturers minutely monitor and control particle size during production to produce consistent batches and meet all relevant regulatory conditions [73].

### 4.5. Blending and Formulation

The blending and formulation step is essential in tailoring HPMC for its various applications, viz. from pharmaceuticals and food to cosmetics and other industrial products. In this step, HPMC is mixed with other ingredients, and the blend is prepared to reach the target desired functional features and properties.

HPMC is seldom used alone. It is very often combined with various excipients, additives, or active ingredients to improve or change its performance. Such admixtures can comprise binders, plasticizers, fillers, disintegrants, flavoring agents, lubricants, or other polymers. The selection of blend components is determined based on the target application. For instance, in pharmaceutical tablet formulations, HPMC could be blended with microcrystalline cellulose or lactose to enhance compressibility and tablet (structural) integrity. In food applications, HPMC might be blended with emulsifiers or stabilizers to adjust texture and improve shelf life. The blend composition is meticulously devised so as to balance properties namely gel strength, viscosity, release profile, and mechanical stability [74]. Where necessary, homogenization is then applied to reduce particle size and increase microstructural uniformity [75]. Optimizing HPMC-based formulations is a step-by-step process that involves testing, adjusting, and refining the mix. HPMC concentration, its particle size, the blend ratios, and processing conditions, are key variables that are varied to examine the resulting effects on product viscosity, (mechanical) strength, gelation temperature, dissolution, and release behavior. Design of Experiments (DoE) can tangibly assist in effectively exploring the effects of these process variables. Tests like rheological analysis, dissolution studies, texture analysis, and stability testing generate the body of specific data that determine the decisions taken to alter HPMC-based formulations towards meeting set target product characteristics, quality standards, works in manufacturing, cost limits, and performance as sought by the end user [22,26,76,77].

### 4.6. Quality Control

Quality control is vital in HPMC production to ensure the final product satisfies all the required standards for its target use. Quality control ensures HPMC is safe, consistent, and reliable for pharmaceutical, cosmetic, food, and industrial applications [78]. The aspects that must be considered for quality are concisely explained below.

#### 4.6.1. Analytical Quantifications and Testing

Pursuant to Section 2.2., quality control for HPMC employs different analytical methods to examine its main properties. Infrared (IR) spectroscopy confirms the chemical structure by identifying the typical absorption bands of ether groups, hydroxyls, and substituents, and brings forward any chemical modifications or degradation. Chromatographic techniques such as HPLC and GC measure the quantities of unreacted reagents, residual solvents, small impurities, and additives. Rheological tests determine viscosity, flow-related properties, and gelation, all of which are central in applications like film formation or controlled release. Other techniques, such as DSC for thermal characteristics, XRD for crystallinity determination, and particle size analysis by microscopy or laser diffraction, also supplement the pool of information needed to realize a more complete and objective quality assessment [26,79].

#### 4.6.2. Determination of the Substitution Pattern

Keeping DSMe and MSHP within their specified limits is essential to ensure that HPMC performs as expected in its final application. Regular and precise measurement of the substitution pattern ensures batch-to-batch consistency and compliance with product specifications. Different analytical methods may be used for this purpose. NMR spectroscopy (see Section 2.2) provides detailed molecular level information on substitution patterns and allows accurate quantification of DSMe. Chemical titration techniques can be used to quantify substituent groups by targeting reactive functional groups. Because the substitution pattern along the HPMC chains strongly influences solution behavior and performance, more advanced liquid-chromatography methods have been developed to characterize HPMC at the molecular level [20]. For instance, Cuers et al. [3], using LC-ESI-MS, examined the substituent distribution along HPMC chains in detail, providing insight into molecular level heterogeneity that goes beyond average substitution values. [3]. In addition, Greiderer et al. [79] characterized HPMC with two-dimensional liquid chromatography (LC × LC), offering much better separation and profiling of complex polymer populations than conventional one-dimensional (LC) [79].

#### 4.6.3. Impurities Monitoring

Ensuring the safety and regulatory compliance of HPMC demands strict monitoring of impurities. The main areas comprise:

Heavy metals: The presence of lead, cadmium, arsenic, and mercury are determined by AAS or ICP-MS. These must absolutely meet pharmacopeial and food-grade safety requirements.

Residual solvents: The levels of residual solvents (e.g., propylene oxide, methyl chloride, or ethanol) used during synthesis or purification are determined by GC or other suitable and adequately sensitive techniques to confirm they are (well) below allowable limits.

Microbial purity: Microbiological tests are conducted to verify that HPMC is free of harmful fungi, bacteria, and endotoxins. Typical tests comprise yeast and mold counts, total aerobic count, endotoxin speciation, and confirmation of the absence of specific pathogens. Sterility testing may be necessary for sterile-grade material.

Other contaminants: Additional verifications of product parameters, such as moisture, ash content and pH, aid in confirming overall purity and stability [79,80].

### 4.7. Packaging and Storage

Suitable packaging and storage are necessary to preserve HPMC’s physicochemical characteristics and maintain its performance for its shelf life. Because HPMC is moisture sensitive, as well as sensitive to other environmental conditions and factors, selecting and using the right packaging materials and defining the appropriate (actually, the optimal) storage conditions become critical steps at this stage [58].

#### 4.7.1. Packaging Materials

HPMC is typically packaged in moisture-resistant and airtight containers to screen it from contamination, humidity, and physical damage [81]. Common packaging routes for HPMC comprise laminated foil bags, multi-layer kraft paper bags with polyethylene liners, rigid plastic containers, and plastic drums. These materials protect the product from oxygen, moisture, and light, which can otherwise induce hydrolytic degradation or alterations in polymer properties. Pharmaceutical grade HPMC may also apply evident seals and must comply with Good Manufacturing Practices (GMPs) to enforce traceability and integrity. Packaging is often developed for easy handling, transportation, and storage, with features such as liners or resealable closures to decrease exposure during use [12].

#### 4.7.2. Storage Conditions

To preserve HPMC quality and stability, storage conditions must be diligently controlled. The polymer should be stored in a dry and cool place, typically below 50% relative humidity and at 15–25 °C. High humidity can induce moisture uptake, agglomeration, poor flow, and even hydrolysis of ether bonds, which wanes performance. Direct sunlight and ultraviolet (UV) exposure should be limited as much as possible because that can result in photodegradation and discoloration. Storage areas should absolutely be clean, free from dust, chemicals, or pests, and be well-ventilated. For long-term storage, climate-controlled installations with humidity and temperature monitoring are highly recommended.

#### 4.7.3. Shelf Life

The shelf life of HPMC depends on its formulation, packaging, and storage conditions. HPMC can preserve its quality and functionality for several months to several years when properly packaged and stored [4]. Its typical shelf lives are 2–5 years, although this can vary subject to the degree of substitution, the moisture level at the time of packaging, and the presence of additives. Manufacturers usually provide a defined expiration date based on stability studies conducted according to regulatory guidelines (e.g., ICH Q1A International Council for Harmonisation of Technical Requirements for Pharmaceuticals for Human Use (ICH) guideline Q1A—“Stability Testing of New Drug Substances and Products”). During the shelf life, periodic quality checks may be performed to monitor parameters such as moisture content, viscosity, and microbial contamination to ensure ongoing compliance. Proper inventory management practices, including first-in-first-out (FIFO) rotation, help minimize the risk of using degraded or expired material [4]. In this way, the manufacturing route and associated quality-control strategy determine whether HPMC meets the structural and functional specifications required for its intended use. Because differences in substitution pattern, viscosity, MW, purity, and particle size translate into differences in hydration, gelation, film formation, and release performance, these production-defined attributes ultimately explain the broad range of HPMC applications. The next section discusses how these material characteristics are exploited in pharmaceutical, food, construction, and other emerging fields. The overall HPMC manufacturing workflow and critical controls across stages 4.1–4.7 are summarized in Table 2.

## 5. Applications of Hydroxypropyl Methylcellulose

HPMC is increasingly recognized for its ecofriendly applications across various industries due to its biodegradable nature and non-toxic properties. Selection of an HPMC grade should be based on specific application needs, including desired viscosity, film-forming ability, water retention, and ingredient compatibility, as well as formulation parameters such as pH, temperature, and processing conditions. For example, high MW HPMC is ideal for applications requiring high viscosity and strong water solubility, such as slow-release pharmaceutical formulations, also providing better film-forming properties and greater resistance to enzyme breakdown. Low MW HPMC is suitable for applications needing lower viscosity and better flow, such as paints and adhesives. The substitution pattern in HPMC refers to the number of natural groups in the cellulose replaced by hydroxypropyl or methyl groups, which affects HPMC’s behavior in various products. Increasing DSMe generally reduces intermolecular hydrogen bonding, which promotes water solubility and relatively high and balanced DSMe and MSHP contribute to thickening, stabilization, or film-forming abilities, which are crucial for applications in construction, food, and pharmaceutical industries [82]. For example, in hot climates where moisture in construction mortar evaporates quickly, HPMC with higher viscosity and better water retention is preferred, while in colder temperatures, HPMC with lower viscosity can be used. HPMC grades utilized in food industry and pharmaceuticals must also meet standards such as the European Pharmacopoeia (Ph. Eur.) and United States Pharmacopeia (USP). These standards clearly define requirements for chemical composition, purity, and performance to ensure effectiveness and safety. As technology progresses, new opportunities for enhancing HPMC’s performance, and its grades are expected to keep developing. Ongoing research is crucial to address current knowledge gaps and optimize HPMC use across different sectors given that formulations can vary by manufacturer and by application [83]. Table 3 summarizes the main actual applications of HPMC, as identified through a critical analysis of the literature.

The versatility of HPMC is closely linked to the possibility of tailoring its functional performance through variations in substitution pattern, MW, viscosity, and particle characteristics. These parameters influence key behaviors such as hydration, water retention, gelation, film formation, thickening capacity, and release control, which in turn determine the suitability of a given HPMC grade for a specific industrial application. Therefore, the wide use of HPMC in construction, food, pharmaceuticals, and other fields is not only a consequence of its cellulose-derived nature but also of the strong relationship between its physicochemical characteristics and its end-use functionality.

Table 4 summarizes how the substitution pattern of HPMC drives its main physicochemical and functional properties. Since DSMe and MSHP determine the polymer’s hydrophilic–hydrophobic balance and its intermolecular interactions, variations in these parameters lead to predictable changes in thermal gelation, viscosity, and water solubility. These shifts ultimately define the most suitable application areas (e.g., immediate- vs. sustained-release drug systems, coatings, food, or cosmetic matrices). Accordingly, Table 5 compares four representative substitution “windows” (low/high DSMe combined with low/high MSHP) and highlights general trends and practical considerations for formulation and grade selection.

### 5.1. Construction Applications

HPMC is a versatile polymer widely used in construction for its water retention, thickening, and adhesive properties. It improves the workability and mechanical performance of cement-based materials like mortars and tile adhesives. In iron ore tailings (IOT) concrete, HPMC increases viscosity and prevents aggregate sinking, making the concrete easier to work with. While it can slightly reduce compressive and flexible strength due to more air bubbles, HPMC improves crack resistance and splitting tensile strength. This reduction is commonly attributed to the air-entraining/thickening effect of HPMC: by increasing slurry viscosity, more air is incorporated and retained during mixing, and the resulting pores after hardening can decrease compressive and flexural strength. Despite increasing the number of air bubbles in concrete, overall strength and durability remain largely unaffected, making HPMC an effective additive for improving the performance of IOT based concrete [84].

Using insulation materials in construction improves energy efficiency by keeping buildings warmer and reducing energy use. HPMC has been studied as an additive in rendering mortars, where it makes the mortar lighter and more porous, improving thermal insulation and reducing heat loss by up to 30%. Higher HPMC concentrations further increase thermal resistance. In general, HPMC contributes to more energy-efficient and sustainable building materials [85].

In construction applications, the choice of HPMC grade is strongly influenced by viscosity and water-retention capacity. Higher viscosity grades are generally preferred in mortars, renders, and tile adhesives because they improve workability, reduce water loss, and help prevent segregation or sedimentation during application. In this way, the performance of HPMC in cement-based systems is directly related to the physicochemical parameters described in the previous sections.

### 5.2. Food Industry Applications

In the food industry, HPMC functions as an extremely versatile ingredient, serving multiple roles such as a stabilizer, thickener, and emulsifier [82]. HPMC’s ability to produce gels and films renders it to be an ideal candidate for ameliorating the moisture retention, texture, and shelf life of food items. Its non-toxic and biodegradable character espouses the growing demand for green, sustainable, and health-conscious food solutions [86]. As for its toxicity, the Food and Drug Administration (FDA) has approved it as both a direct and indirect food additive, and it is also sanctioned for use as a food additive by the European Union. The Joint FAO/WHO Expert Committee on Food Additives (JECFA) has assessed HPMC’s applications in food and determined an acceptable daily intake (ADI) of ‘not specified’, indicating no specific limit is necessary. From a 90-day feeding study in rats, a no-observed-adverse-effect level (NOAEL) of 5000 mg/kg body weight/day was identified. Consequently, a tolerable intake for humans is suggested at 5 mg/kg body weight/day, which is over 100 times higher than the current estimated consumption of 0.047 mg/kg body weight/day [14].

Takin into account the different applications in the food industry, HPMC serves as a vegetarian alternative to gelatin in various food products [87]. It is used in the production of edible films and coatings, which are biodegradable and safe for consumption.

In food systems, the performance of HPMC depends largely on its substitution pattern and viscosity, since these parameters govern thickening behavior, emulsion stabilization, texture development, and thermal gelation. As a result, the selection of a specific HPMC grade is closely related to the functional requirement of the product, whether as a stabilizer, film former, or texturizing agent.

### 5.3. Pharmaceutical Application

HPMC plays a great role in the pharmaceutical industry due to its multifunctional properties and versatility [88]. HPMC’s ability to enhance the solubility and bioavailability of active pharmaceutical ingredients makes it an essential component in the development of effective and patient-friendly medications [89]. Additionally, its non-toxic and biocompatible nature makes it suitable for pharmaceutical applications and consistent with the principles of green chemistry. This section reviews the main pharmaceutical applications of HPMC, highlighting its use as a binder, film-forming agent, and controlled-release excipient in drug formulations, as well as its role in improving drug delivery and therapeutic performance [90,91].

As already stated (please see Section 2.2.7), HPMC typically has a Tg of 170–180 °C and forms a gel when heated above 75–90 °C [17]. This property makes HPMC suitable for use as capsule shells when substituting gelatin [2]. However, the dissolution and disintegration of HPMC capsules decrease when the temperature exceeds 30 °C, so it is recommended to take these capsules with cold water. Compared to hard gelatin, HPMC capsules are more widely accepted by consumers due to their vegetable-derived ingredients [92].

Kadry et al. [92] used HPMC and diltiazem to make filaments for 3D-printed tablets with different designs. They found that the tablet structure affected how quickly the drug was released—higher infill density slowed release, while alternating drug-free and drug-loaded layers created delayed or intermittent release. The drug absorption in rats matched these release patterns, showing the potential to control drug delivery using 3D printing [92].

Plasticized high MW HPMC (USP2208) improves tablet formation by enhancing compression and compaction properties. Among tested plasticizers, propylene glycol (PG) was the most effective, decreasing the Tg, raising plasticity, and enhancing tablet strength at lower pressures. Other plasticizers such as dibutyl sebacate, glycerol and triacetin proved less effective, often producing weaker tablets and more expansion. The study by Hardy et al. shows that PG improves HPMC tablet formation by encouraging intermolecular bonding through hydroxyl groups, while hydrophobic plasticizers do not offer this benefit [93].

A comprehensive overview of the use of 3D printing technology in the pharmaceutical industry by Muehlenfeld et al. [29] discusses 3D printing technology usage in the pharmaceutical industry by covering the formulation of drug-loaded filaments, the development of sustained-release matrix tablets and process monitoring tools such as in-line Raman spectroscopy. The review also discusses the challenges linked with thermally sensitive drugs and emerging strategies (e.g., foam-based extrusion). 3D printing allows the production of drug-loaded filaments, sustained-release tablets, and specialized coatings designed for extended drug release. The technology uses tools like in-line Raman spectroscopy for process monitoring and explores methods such as supercritical CO_2_ foam extrusion. While 3D printing offers many innovative ways to make medicines, it also faces challenges, especially with heat-sensitive drugs. In general, 3D printing shows great promise for transforming how medicines are made and delivered [29].

A study developed matrix tablets using indomethacin, PEG, and HPMC to improve the drug’s solubility and control its release. HPMC slowed the drug release, while PEG increased solubility. The results showed that the PEG-HPMC system is effective for making controlled-release tablets, which could be useful for other drugs with low solubility [94].

The rheological characteristics of HPMC solutions highlight their eco-friendly applications, particularly in ophthalmic formulations. Increasing the gradient decreases the viscosity of HPMC solutions. Solutions with 0.25% and 0.5% concentrations exhibit non-Newtonian flows at low gradients up to 100 s^−1^, transitioning to Newtonian fluids as the gradient increases. A 1% HPMC solution behaves similarly, with non-Newtonian flow observed up to 250 s^−1^. The correlation between solution viscosity and HPMC concentration at a gradient of 437 s^−1^ is evident. A 2% HPMC solution demonstrates a pseudoplastic flow, with viscosity increasing threefold from 0.25% to 0.50% concentration, and fivefold from 0.50% to 1.00%, reaching 0.087 Pa.s. This exponential viscosity increase enhances retention on the cornea, improving contact but potentially blurring vision due to high viscosity. The lacrimal fluid viscosity of a healthy eye range between 1 and 10 cP. The correlation between viscosity changes and HPMC concentration at 437 s^−1^. Surface tension measurements of HPMC solutions, using an interface tensiometer based on the “ring-method,” were compared to commercial eye drops Indocollyre^®^ at 35 °C. These findings demonstrate HPMC’s potential to be an eco-friendly polymer for sustainable pharmaceutical applications [95].

The pharmaceutical manufacturing process is complex and highly regulated and thus carries its very own specific challenges. The industry involves the design, development, and production of medicinal products, supported by an intricate supply chain because of its highly specialized procurement, manufacturing, and storage requirements. Supply chain challenges often pertain to quality, costs, and regulatory compliance, whereas research and development (R&D) have to deal with formulation needs and patient-centered considerations. Effective coordination between R&D and procurement is thus essential, because both have significant bearings on the manufacturability and marketability of pharmaceutical products. A study using the Analytical Hierarchy Process (AHP) to support R&D decision-making revealed the difficulties in integrating criteria like regulatory constraints and costs. The research by Akbal Dağistan et al. [96] assessed different excipients for oral dispersible films and found HPMC to be the most suitable polymer because of its strong film-forming ability, satisfactory moisture profile, and appropriate viscosity. The authors emphasized the need for a general decision-making tool that could factor in both R&D and supply-chain requirements, which, in turn, result in robust formulations and reliable products. Modern manufacturing principles, known for their shorter product life cycles and the demand for sustainable practices, further emphasize the significance of coordinated and integrated research approaches for the pharmaceutical industry [96].

In pharmaceutical formulations, the versatility of HPMC is especially evident because relatively small differences in MW, viscosity, and substitution pattern can lead to major changes in hydration, gel-layer formation, mucoadhesion, and drug-release behavior. For this reason, grade selection is critical when HPMC is used as a binder, film-forming agent, matrix former, capsule material, or excipient in controlled-release systems.

### 5.4. Other Applications

In addition to its well-established roles in the construction, food, and pharmaceutical industries, HPMC is garnering more attention for its versatility in diverse emerging and technologically advanced applications. Its unique combination of physicochemical properties and biocompatibility [33] and biodegradability has enabled researchers and industry professionals to probe novel applications that reach out well beyond traditional sectors [97]. Recent studies have demonstrated HPMC’s potential in areas like green chemistry, advanced materials engineering, environmental protection, and additive manufacturing. These emerging applications both emphasize HPMC’s remarkable adaptability and its value as a sustainable and functional material in today’s science and industry [7,98].

Additional studies have demonstrated that HPMC is an effective and sustainable additive in water-based (green) chemistry. It accelerates reaction rates and can form gel-like structures and hydrophobic pockets for nanoparticles, thus rendering some transformations more efficient and more rapid. HPMC is particularly useful in amidation and amination chemistries, as well as in reducing waste and simplifying procedures, and in forming capsules for metal-catalyzed processes. Together, these findings demonstrate that HPMC aligns itself with greener chemical synthesis [7].

The study by Polamaplly et al. [99] showed that HPMC and methylcellulose (MC) can serve as biodegradable support materials in 3D printing, thus assisting in the synthesis of complex geometries alongside reducing environmental impacts. These cellulose-based hydrogels are endowed with excellent printability, therefore offering a greener option to petroleum-based supports (e.g., acrylonitrile butadiene styrene). Using biodegradable supports helps lower toxic waste production and encourages more sustainable 3D-printing practices [29].

In Zhu et al.’s study [100], it has been shown that incorporating HPMC into sand treated with MICP technology increased its strength and resistance to erosion by improving calcium carbonate formation. HPMC also helped retain ammonia, thereby decreasing its release into the environment. When combined with MICP, HPMC decreases erosion, improves soil stability, and minimizes ammonia emissions. XRD and SEM analyses confirmed these effects, thus validating HPMC as an effective additive for sustainable soil stabilization and environmental protection [100].

Recent studies have further pointed to the broad applicability of HPMC in advanced functional materials. Giang et al. [18] investigated how different additives influenced the fabrication and features of HPMC-based hydrogels, showing side-by-side that formulation variables could considerably affect their structural and performance. These hydrogels readily pose as promising candidates for pharmaceutical and biomedical applications by virtue of their biodegradability, potential biocompatibility, and physicochemical behaviors. Wang et al. [101] probed the effect of surfactants on polymer-maintained nifedipine supersaturation in aqueous media. Therein, it was found that the type of surfactant and its interaction with polymers, such as HPMC, could either ameliorate or hamper the maintenance of drug supersaturation. These findings clearly highlight the relevance of thorough formulation design when HPMC is included in drug-delivery systems.

## 6. Environmental Impact of Hydroxypropyl Methylcellulose and Life Cycle

### 6.1. Main Environmental Impacts

The environmental impact assessment of HPMC production includes many factors, inter alia, gaseous emissions, energy consumption, waste generation, and its biodegradability and toxicity profiles [13]. A summary is provided in Table 5.

HPMC production is relatively energy-intensive, particularly because of the etherification and drying stages, and its footprint is also influenced by the origin and processing of cellulose feedstocks [10]. Although HPMC is not classified as “*readily biodegradable*” under standard OECD testing, many studies indicate that it can biodegrade given the conducive environmental conditions and biodegrading usually faster than most synthetic polymers [102]. Together with its low toxicity, this suggests a comparatively low environmental hazard, although disposal in large amounts still requires appropriate management [103,104].

From an industrial perspective, relevant impacts also arise from VOC emissions during etherification, transport-related emissions, and the generation of solid and liquid residues, including off-specification products, spent process materials, filtration residues, and packaging waste [105].

In practice, HPMC waste is generally managed within broader EHS and industrial waste-management frameworks rather than under HPMC-specific legislation. Therefore, appropriate handling of polymer-containing effluents and solid residues remains important to minimize accumulation and environmental release, especially in cases of process losses, washing streams, or rejected batches [30,106,107].

### 6.2. Life Cycle Assessment (LCA) and Environmental Product Declarations (EPD)

Life cycle assessment (LCA) is a standardized methodology for evaluating the environmental impact of a product, process, or service from raw material acquisition through to end of life. LCA provides objective, quantitative insight into the environmental footprint of each stage, as a result supporting take a decision for greater sustainability [8]. In cellulose-based systems, previous studies have shown that environmental hotspots are commonly linked to upstream raw material processing, chemical use, energy demand, and end-of-life scenarios, with results being sensitive to methodological choices such as system boundaries and allocation criteria.

For HPMC specifically, dedicated academic LCA studies are still lacking. Therefore, the main publicly available HPMC-specific environmental information currently comes from manufacturer-issued Environmental Product Declarations (EPDs). These documents generally follow ISO 14025 and ISO 14040 principles and are typically structured as cradle-to-gate assessments based on a functional unit of 1 kg of product. Available EPD data indicate that major contributors to global warming potential include methyl chloride, propylene oxide, sodium hydroxide, and process energy demand, while additional impacts are associated with transport and end-of-life stages [102]. Table 6 depicts the LCA results of the corresponding published EPDs for a HPMC produced from cotton linters [13].

## 7. Conclusions

Hydroxypropyl methylcellulose (HPMC) remains one of the most versatile and high-value polymers for the pharmaceutical, food, and construction industrial sectors. As a semi-synthetic polymer obtained from renewable cellulosic feedstocks, HPMC is considered to have a lower environmental impact than many synthetic polymers based on non-renewable fossil resources. Its physicochemical properties, including thermal gelation, variety of viscosity, and film-forming capacity—together with its biocompatibility and biodegradability—enable a wide spectrum of technological applications, from oral controlled-release systems and topical formulations to coatings, binders, and structural modifiers in construction and food matrices. The literature shows that the choice of grade, MW, particle size, and substitution pattern plays a critical role in determining its performance for different functions, such as solubility enhancement, bioavailability optimization, water retention, stabilization, and mechanical reinforcement.

Recent advances in analytics and process engineering have increased the understanding of HPMC functionality, enabling a more rational and targeted selection of material attributes. Nonetheless, the variability associated with raw material sources and processing routes remains a significant challenge for the industry, reinforcing the need for robust quality control.

Presently, the applications of HPMC are broad and extended, mainly as additive in construction, food, and pharmaceutical industries, as well as in emerging applications such as technological and chemical synthesis sectors. Thus, the use of HPMC is not expected to decrease in the near future but rather to grow.For this reason, it is essential to ensure regulatory alignment together with cleaner and greener processes along its life cycle to reduce HPMC’s environmental impact. Specifically, responsible sourcing of cellulose, improved control of substitution and purification processes, reduced solvent and reagent consumption, lower emissions, higher energy efficiency, and better end-to-end life cycle assessment are necessary to be implemented.

HPMC is strategically positioned as a promising semi-synthetic polymer for the development of greener materials and products. To further harness its versatility and address current industrial challenges, future research should focus on:

- Deeper understanding of the relationships between substitution patterns, molecular architecture, and end-use performances.
- More standardized comparisons among commercial HPMC grades to ensure predictability in formulations.
- Improved knowledge of processing–structure–property relationships across different industrial contexts.
- Sustainability metrics, greener production routes, and comprehensive life cycle assessments.
- Advanced applications of HPMC in functional materials, active packaging, controlled release systems, and sustainable formulations.

## Figures and Tables

**Table 1:** Commercial grades of various types of HPMC based on viscosity and USP specifications, categorized based on their levels of hydroxypropyl and methoxy substitutions with their properties and applications in construction, food and pharma industries (adapted from [1]).

USP	Grade	Viscosity (cP)	Methoxy (%)	Hydroxypropyl (%)	Properties	Construction	Food	Pharma
2910	E 3 Premium LV	3	28–30	7–12	Low viscosity, fast hydration, clear solution	Tile adhesives, wall putty, cement renders	Food emulsifier, stabilizer	Tablet film coating, binder
2910	E 5 Premium LV	5	28–30	7–12	Low viscosity, easily dispersible	Paints, plasters, joint fillers	Bakery product texture, sauce thickener	Tablet film coating, binder
2910	E 6 Premium LV	6	28–30	7–12	Low–medium viscosity, stabilizer	Gypsum and cement plasters, renders	Dairy dessert stabilizer, ice cream	Sustained-release agent, granulation binder
2910	E 15 Premium LV	15	28–30	7–12	Moderate viscosity, good film-former	Wall skim coat, repair mortars, putty	Dairy thickener, cream stabilizer	Film coating, capsule production
2910	E 50 Premium LV	50	28–30	7–12	Moderate viscosity, thickening and binding	Cement renders, mortars, adhesives	Food sauce and soup thickener	Sustained-release matrix, film coating
2910	E 4M Premium	4000	28–30	7–12	High viscosity, gel forming, water retention	Tile adhesives, EIFS, self-leveling compounds	Bread improver, texture modifier	Controlled-release agent, matrix design
2910	E 4M Premium CR	4000	28–30	7–12	High viscosity, controlled rheology	Tile adhesives, repair mortars	Meat binder, emulsifier	Modified release coatings, hydrophilic matrix
2910	E 10M Premium CR	10,000	28–30	7–12	Very high viscosity, gel structure	Self-leveling compounds, tile adhesives	Gel desserts, food thickeners	Extended-release matrices
2906	F 4M Premium	4000	27–30	4–7.5	High viscosity, lower hydroxypropyl	Plaster and putty improvement	Frozen dough stabilizer, emulsifier	Binder in direct compression, film coating
2208	K 3 Premium LV	3	19–24	4–12	Low viscosity, good solubility	Self-level flooring, sprayable mortars	Fat replacer, beverage clouding agent	Artificial tears, ophthalmic lubricants
2208	K 100 Premium LV	100	19–24	4–12	Medium viscosity, effective thickener	Cement tile adhesives, grout, putty	Ice cream stabilizer and thickener	Tablet binder, controlled release
2208	K 100 Premium LV CR	100	19–24	4–12	Medium viscosity, controlled rheology	Skim coat mortar, brick mortars	Food suspending agent, beverage stabilizer	Controlled-release matrix, film formation
2208	K 100 Premium LV LH	100	19–24	4–12	Medium viscosity, low hydroxypropyl	Smoothing compounds, repair mortars	Milk and cream foaming agent	Sustained-release matrix, capsule coating
2208	K 100 Premium LV LH CR	100	19–24	4–12	Medium viscosity, slow-release properties	Leveling compounds, masonry mortars	Emulsifier and extender in foods	Extended-release agent, oral suspensions
2208	K 4M Premium	4000	19–24	4–12	High viscosity, excellent water retention	Mortars, grouts, tile adhesives	Frozen dessert stabilizer, bread dough enhancer	Hydrophilic matrix, binder in tablets
2208	K 4M Premium CR	4000	19–24	4–12	High viscosity, controlled rheology	Repair mortars, high thixotropy adhesives	Meat product binder, processed food thickener	Controlled-release, matrix for osmotic tablets
2208	K 15M Premium	15,000	19–24	4–12	Very high viscosity, strong thickener	Joint compounds, thick render pastes	Dough improver for bakery applications	Modified-release tablet cores, binder
2208	K 15M Premium CR	15,000	19–24	4–12	Very high viscosity, controlled release	Tile adhesives, grouting	Emulsifier and texturizer in processed foods	Matrix agent for extended-release tablets
2208	K 100M Premium	100,000	19–24	4–12	Ultra-high viscosity, highly water-retentive	Highly thixotropic plasters, adhesives	Food gelling agent, dessert gels	Hydrophilic matrix, texturizer for pharmaceuticals
2208	K 100M Premium CR	100,000	19–24	4–12	Ultra-high viscosity, modified rheology	Grout and mortar additives, repair mortars	Food texture modifier, stabilizer in gels	Controlled-release matrix, capsule gelling agent

**Table 2:** Manufacturing process stages with key parameters (original compilation based on the literature cited in this section).

Stage	Purpose	Inputs	Key Process Controls	Output	Main Quality Risks
**Raw Material Selection** 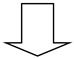	Selection of cellulose source and confirmation of purity for consistent reactivity and final performance	Cotton linters or wood pulp; incoming specs/CoA	Source qualification; impurity limits (lignin/hemicellulose/ash); incoming QC	Qualified cellulose feedstock	Variability in starting cellulose affects DS/MW and downstream reproducibility
**Etherification Reaction** 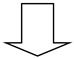	Chemical modification of cellulose to introduce methoxy and hydroxypropyl substituents (HPMC formation)	NaOH/KOH (alkalization); methyl chloride; propylene oxide	Temperature, pressure, pH (alkaline), time; in-process sampling (DSMe, MSHP, MW)	Etherified HPMC reaction mass	Under-/over-substitution; degradation/crosslinking; residual reagents/by-products
**Removal of Impurities (Purification and Washing)** 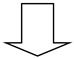	Removal of soluble residues, salts, catalysts, residual reagents, and low-MW by-products; conditioning	Water; possible alcohols (ethanol/isopropanol); pH adjustment agents (as needed)	Washing cycles; solid–liquid separation efficiency; pH/conductivity; residuals tracking	Purified wet HPMC cake	Insufficient washing → residual solvents/salts; product losses; inconsistent purity
**Filtration and Drying** 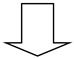	Separation from wash liquor and controlled drying to stable powder/granules	Filtration aids (if used); drying air/energy	Filtration mode (vacuum/pressure/centrifuge); drying temperature/time; residual moisture/solvent	Dried HPMC	Over-drying/thermal stress; residual moisture → clumping/instability
**Particle Size Control** 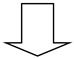	Milling and classification to meet target PSD and handling/performance	Dried HPMC; milling/sieving equipment	Milling parameters (speed/feed/time/heat); sieve cuts; PSD testing (laser diffraction/microscopy)	HPMC with specified particle size distribution	Too fine → dusting; too coarse → slow hydration; PSD variability affects performance
**Blending and Formulation** 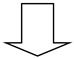	Homogenization and tailoring (when applicable) to meet application specs	HPMC + excipients/additives/actives (as relevant)	Mixing time/speed/order; blend uniformity; DoE-based optimization (as needed)	Homogeneous blend/formulated product	Segregation; inconsistent functionality; interaction effects (e.g., viscosity/release)
**Quality Control** 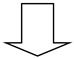	Analytical verification of identity, substitution pattern, impurities, and performance attributes	QC methods (IR, NMR, GC/HPLC, rheology, DSC, XRD, PSD, microbiology, ICP-MS)	Release specs: DSMe/MSHP, viscosity, MW, residual solvents, metals, microbes, moisture, etc.	Released batch (compliant)	Non-compliance with pharmacopeias/food regs; batch-to-batch drift
**Packaging and Storage**	Protection from moisture/contamination; stability through shelf life	Moisture barrier packs (bags/drums/liners); labels	RH/temperature control; sealing integrity; FIFO; stability checks	Packaged HPMC with defined shelf life	Moisture uptake → caking/property changes; contamination; label/traceability issues

**Table 3:** Applications of HPMC in the different industrial sectors together the respective research gaps and challenges.

Key Observations	Sector	Research Gaps and Challenges
- Variability in DS and MW affects performance. - Need for greener synthesis methods.	**General**	- Optimization of HPMC grades for specific applications. - Standardization between manufacturers. - Regulatory compliance (USP, Ph. Eur.). - Improvements in sustainability and recycling. - Optimization of functional properties (water retention, film formation, etc.).
- Improves workability and durability. - May reduce compressive strength due to air-entrainment.	**Construction**	- Balancing workability and mechanical strength. - Minimizing air-entrainment effects on strength. - Optimizing formulations for extreme conditions (climate, humidity). - Integration into new composites (MgO, Fe_2_O_3_).
- Vegetarian alternative to gelatin. - Approved by FDA and EU. - Applications in active packaging.	**Food**	- Optimizing mechanical and barrier properties in biodegradable films. - Development of intelligent packaging (pH indicators). - Compliance with food safety regulations.
- Essential in sustained-release formulations. - Increasing use of plant-based capsules.	**Pharma**	- Optimizing controlled-release profiles. - Improving compaction and matrix properties. - Integration with advanced technologies (3D printing). - Understanding polymer–surfactant interactions.
- Potential in green chemistry and biodegradable materials. - Enhances erosion resistance in soils.	**Other**	- Improving tribological and green lubrication properties. - Optimizing crosslinking methods and additives. - Assessing environmental impact on soil stabilization applications.

**Table 4:** Properties of hydroxypropyl methylcellulose based on its substitution pattern (values represent typical ranges reported in the literature [16,17,24,34]).

Parameter/Degree of Substitution	Low DSMe/Low MSHP	High DSMe/Low MSHP	Low DSMe/High MSHP	High DSMe/High MSHP	General Remarks
Substituent Groups	Few methoxy, few hydroxypropyl	Many methoxy, few hydroxypropyl	Few methoxy, many hydroxypropyl	Many methoxy, many hydroxypropyl	DSMe—methoxy degree of substitution (typical 1.3–2.1); MSHP—hydroxypropyl molar (typical 0.1–1.0)
					
Viscosity	Low	Medium	Medium	High	Increases with MW and substitution; commercial grades of 3–100,000 cP
					
Thermal Gelation	High gelation temperature	Low gelation temperature.	Medium gelation temperature.	Low gelation temperature.	Higher DSMe and lower MSHP = lower gelation temperature
					
Water Solubility	High	Medium	Very high	Medium	Higher MSHP/DSMe favors solubility; low viscosity = higher solubility
					
Organic Solubility	Very low	Very low	Very low	Very low	Insoluble in acetone, chloroform, etc.
					
Applications	Fast drug release, coatings	Controlled-release matrices, ophthalmic gels	Hydrophilic matrices, food, cosmetics	Sustained-release systems, adhesives	Viscosity and gelation determine use in pharma, food, cosmetics
					
Environmental Impact	Biodegradable, low impact	Biodegradable, low impact	Biodegradable, low impact	Biodegradable, low impact	Cellulose-derived, non-toxic, low environmental risk

**Table 5:** Environmental impact of hydroxypropyl methylcellulose along the different stages of its life cycle (original summary and compilation of the relevant literature).

Life-Cycle Stage	Description	Environmental Impact/Remarks
Raw Material Extraction	Cellulose source: the main raw material for HPMC is cellulose, typically derived from cotton or wood.	Impacts: deforestation, energy consumption, and emissions from harvesting and processing cellulose.
		
Production	Chemical processing: HPMC production involves chemical reactions, including etherification.	Impacts: use of chemicals and energy, with significant emissions.
	Energy use: the manufacturing process is energy intensive.	Impacts: contribution to greenhouse gas emissions.
Transport	Logistics: the transport of raw materials to the production plant and the distribution of the final product to consumers.	Impacts: fuel consumption and emissions associated with transportation.
		
Use	Application: in its various applications, HPMC generally has a low environmental impact.	Note: specific use may influence the overall environmental footprint.
		
End of Life	Disposal: the biodegradability of HPMC is a positive factor, but disposal methods (landfill, incineration) can generate impacts.	Impacts: depending on the disposal method, environmental effects may occur.
	Sustainability efforts: companies aim to reduce environmental impact by improving energy efficiency, using sustainable raw materials, and optimizing processes.	Note: growing trend towards sustainability in HPMC production.
	Regulatory compliance: complying with environmental regulations and standards is essential to minimize the ecological footprint.	Note: regulatory compliance helps to reduce the environmental impact of HPMC production.

**Table 6:** Life cycle stage breakdown of environmental impacts of a hydroxypropyl methylcellulose produced from cotton linters (per 1 kg product, including packaging, cradle-to-grave). Source: ETON-AM [13].

Indicator	Unit	Upstream	Core	Downstream	Total
Global Warming Potential (total)	kg CO_2_	3.75	4.81	2.35	10.9
GWP—Fossil	kg CO_2_	6.06	4.78	0.349	11.2
GWP—Biogenic	kg CO_2_	−2.31	0.0287	2.00	−0.286
GWP—LULUC	kg CO_2_	0.00493	0.00135	0.000181	0.00647
Ozone Depletion Potential (ODP)	kg CFC11	3.77 × 10^−5^	3.39 × 10^−8^	4.75 × 10^−9^	3.77 × 10^−5^
Acidification Potential (AP)	mol H^+^	0.0591	0.0211	0.00148	0.0816
Eutrophication (freshwater, EP)	kg P	0.0105	0.000755	0.0000297	0.0113
Eutrophication (marine, EP)	kg N	0.0697	0.00438	0.00229	0.0764
Eutrophication (terrestrial, EP)	mol N	0.182	0.0434	0.00529	0.231
Photochemical Ozone Creation (POCP)	kg NMVOC	0.0258	0.0136	0.00235	0.0417
Abiotic Depletion—Minerals/Metals (ADP-M&M)	kg Sb	2.39 × 10^−5^	2.30 × 10^−6^	1.04 × 10^−6^	2.72 × 10^−5^
Abiotic Depletion—Fossil (ADP-fossil)	MJ	75.5	51.3	4.32	131
Water Deprivation Potential (WDP)	m^3^	2.63	−1.24	0.0297	1.42
Renewable Primary Energy (PERE)	MJ	0	1.04	0.0345	1.07
Renewable Mat. Energy (PERM)	MJ	28.8	0	0	28.8
Non-Renewable Primary Energy (PENRT)	MJ	75.5	51.3	4.32	131
Net Freshwater Use (FW)	m^3^	0	0.011	0	0.011
Hazardous Waste Disposed (HWD)	kg	0	0.00395	0	0.00395
Non-Hazardous Waste Disposed (NHWD)	kg	0	0.417	1.00	1.42
Radioactive Waste Disposed (RWD)	kg	0	0	0	0
Particulate Matter (PM)	disease inc.	7.00 × 10^−7^	2.87 × 10^−7^	2.42 × 10^−8^	1.01 × 10^−6^
Ionizing Radiation (IRP)	kBq U235	0.264	0.0965	0.00511	0.366
Ecotoxicity—Freshwater (ETP-fw)	CTUe	179	29.5	6.68	216
Human Toxicity—Cancer (ETP-c)	CTUh	2.79 × 10^−10^	1.01 × 10^−10^	1.51 × 10^−10^	1.44 × 10^−9^
Human Toxicity—Non-Cancer (HTP-nc)	CTUh	−9.31 × 10^−8^	3.36 × 10^−8^	6.21 × 10^−9^	−5.33 × 10^−8^
Land Use/Soil Quality (SQP)	dimensionless	548	7.55	2.80	558

## Data Availability

No new data were created or analyzed in this study.

## Supplementary Materials

The following supporting information can be downloaded at: https://www.mdpi.com/article/10.3390/polym18091105/s1, Table S1: Analytical techniques for the characterization of HPMC together with the information provided by each technique and examples of practical insights.

## References

[1] Vlad R.-A., Pintea A., Pintea C., Rédai E.-M., Antonoaea P., Bîrsan M., Ciurba A. (2025). Hydroxypropyl Methylcellulose—A Key Excipient in Pharmaceutical Drug Delivery Systems. Pharmaceutics.

[2] Li C.L., Martini L.G., Ford J.L., Roberts M. (2005). The Use of Hypromellose in Oral Drug Delivery. J. Pharm. Pharmacol..

[3] Cuers J., Rinken M., Adden R., Mischnick P. (2013). Critical Investigation of the Substituent Distribution in the Polymer Chains of Hydroxypropyl Methylcelluloses by (LC-)ESI-MS. Anal. Bioanal. Chem..

[4] Niazi S.K. (2004). Stability Testing of New Drug Substances and Products. Handbook of Pharmaceutical Manufacturing Formulations.

[5] Shi J., Sheng L., Chen M., Wu X., Li X., Tan Y.Q. (2024). PH-Responsive Collagen Nanocomposite Films Reinforced by Curcumin-Loaded Laponite Nanoplatelets for Dynamic Visualization of Shrimp Freshness. Food Hydrocoll..

[6] Shi J., He J., Sheng L., Wu X., Mao S., Zhang Y., Xiang C., Sun L. (2026). Stimuli-Responsive Multicolor Nacre-Mimetic Phosphorescent Bionanocomposite Thin Films via Network-Confinement Coupling. Adv. Mater..

[7] Mobilawon F.E., Iroegbu A.O.C., Zinyemba O., Meijboom R. (2025). Hydroxypropyl Methylcellulose (HPMC) in Sustainable Pharmaceutical Synthesis—Mechanistic Insights, Green Metrics and Outlook. Sustain. Chem. Pharm..

[8] Gadaleta G., Ferrara C., De Gisi S., Notarnicola M., De Feo G. (2023). Life Cycle Assessment of End-of-Life Options for Cellulose-Based Bioplastics When Introduced into a Municipal Solid Waste Management System. Sci. Total Environ..

[9] Kaynak E., Piri I.S., Das O. (2025). Revisiting the Basics of Life Cycle Assessment and Lifecycle Thinking. Sustainability.

[10] Onwukamike K.N., Grelier S., Grau E., Cramail H., Meier M.A.R. (2019). Critical Review on Sustainable Homogeneous Cellulose Modification: Why Renewability Is Not Enough. ACS Sustain. Chem. Eng..

[11] Abolore R.S., Jaiswal S., Jaiswal A.K. (2024). Green and Sustainable Pretreatment Methods for Cellulose Extraction from Lignocellulosic Biomass and Its Applications: A Review. Carbohydr. Polym. Technol. Appl..

[12] Ibrahim I.D., Hamam Y., Sadiku E.R., Ndambuki J.M., Kupolati W.K., Jamiru T., Eze A.A., Snyman J. (2022). Need for Sustainable Packaging: An Overview. Polymers.

[13] EPD International AB Hydroxypropyl Methylcellulose (HPMC) Produced in Europe. https://www.environdec.com/library/epd13216.

[14] Burdock G.A. (2007). Safety Assessment of Hydroxypropyl Methylcellulose as a Food Ingredient. Food Chem. Toxicol..

[15] Amanzholkyzy A., Zhumagaliyeva S., Sultanova N., Abilov Z., Ongalbek D., Donbayeva E., Niyazbekova A., Mukazhanova Z. (2025). Hydrogel Delivery Systems for Biological Active Substances: Properties and the Role of HPMC as a Carrier. Molecules.

[16] Perez-Robles S., Carotenuto C., Minale M. (2022). Effect on the Thermo-Gelation Process of the Degree and Molar Substitution of HPMC Polymer Hydrogels. Macromol. Symp..

[17] Svoboda R., Nevyhoštěná M., Macháčková J., Vaculík J., Knotková K., Chromčíková M., Komersová A. (2023). Thermal Degradation of Affinisol HPMC: Optimum Processing Temperatures for Hot Melt Extrusion and 3D Printing. Pharm. Res..

[18] Giang H.N., Le A.T.K., Huynh T.N.A., Phung T.K., Sakai W. (2023). Effect of Additives on Fabrication and Properties of Hydroxypropyl Methylcellulose-Based Hydrogels. Polym. Bull..

[19] Larsson M., Viridén A., Stading M., Larsson A. (2010). The Influence of HPMC Substitution Pattern on Solid-State Properties. Carbohydr. Polym..

[20] Viridén A., Wittgren B., Andersson T., Abrahmsén-Alami S., Larsson A. (2009). Influence of Substitution Pattern on Solution Behavior of Hydroxypropyl Methylcellulose. Biomacromolecules.

[21] Li Y., Shen H., Lyons J.W., Sammler R.L., Brackhagen M., Meunier D.M. (2016). Size-Exclusion Chromatography of Ultrahigh Molecular Weight Methylcellulose Ethers and Hydroxypropyl Methylcellulose Ethers for Reliable Molecular Weight Distribution Characterization. Carbohydr. Polym..

[22] Knarr M., Rogers T.L., Petermann O., Adden R. (2025). Investigation and Rank-Ordering of Hydroxypropyl Methylcellulose (HPMC) Properties Impacting Controlled Release Performance. J. Drug Deliv. Sci. Technol..

[23] Sakkal M., Arafat M., Yuvaraju P., Beiram R., AbuRuz S. (2024). Preparation and Characterization of Theophylline Controlled Release Matrix System Incorporating Poloxamer 407, Stearyl Alcohol, and Hydroxypropyl Methylcellulose: A Novel Formulation and Development Study. Polymers.

[24] Goldoozian S., Mohylyuk V., Dashevskiy A., Bodmeier R. (2021). Gel Strength of Hydrophilic Matrix Tablets in Terms of In Vitro Robustness. Pharm. Res..

[25] HPMC Factory Technical Guidelines for Hydroxypropyl Methylcellulose (HPMC). https://www.hpmcfactory.com/technical-guidelines-for-hydroxypropyl-methylcellulose-hpmc.html.

[26] Saepang K., Pitaksuteepong T., Buranrat B., Boontha S. (2024). Optimization of HPMC-Based Oral Fast Dissolving Film of Cetirizine Dihydrochloride. Nat. Life Sci. Commun..

[27] DuPont Polymers for Industrial Applications METHOCEL^TM^ Water-Soluble Cellulosic Polymers for Industrial Applications. https://cms.chempoint.com/ChemPoint/media/Methocel-brochure/Methocel-Brochure-2019-1.pdf.

[28] De Simone V., Dalmoro A., Lamberti G., Caccavo D., d’Amore M., Barba A.A. (2019). Effect of Binder and Load Solubility Properties on HPMC Granules Produced by Wet Granulation Process. J. Drug Deliv. Sci. Technol..

[29] Muehlenfeld C., Duffy P., Yang F., Zermeño Pérez D., El-Saleh F., Durig T. (2024). Excipients in Pharmaceutical Additive Manufacturing: A Comprehensive Exploration of Polymeric Material Selection for Enhanced 3D Printing. Pharmaceutics.

[30] Allenspach C., Timmins P., Sharif S., Minko T. (2020). Characterization of a Novel Hydroxypropyl Methylcellulose (HPMC) Direct Compression Grade Excipient for Pharmaceutical Tablets. Int. J. Pharm..

[31] Shetty G.R., Rao B.L., Asha S., Wang Y., Sangappa Y. (2015). Preparation and Characterization of Silk Fibroin/Hydroxypropyl Methyl Cellulose (HPMC) Blend Films. Fibers Polym..

[32] Akinosho H., Hawkins S., Wicker L. (2013). Hydroxypropyl Methylcellulose Substituent Analysis and Rheological Properties. Carbohydr. Polym..

[33] Filimon A., Onofrei M.D., Bargan A., Stoica I., Dunca S. (2023). Bioactive Materials Based on Hydroxypropyl Methylcellulose and Silver Nanoparticles: Structural-Morphological Characterization and Antimicrobial Testing. Polymers.

[34] Wan S., Dai C., Bai Y., Xie W., Guan T., Sun H., Wang B. (2021). Application of Multivariate Methods to Evaluate Differential Material Attributes of HPMC from Different Sources. ACS Omega.

[35] Malatesta M. (2021). Transmission Electron Microscopy as a Powerful Tool to Investigate the Interaction of Nanoparticles with Subcellular Structures. Int. J. Mol. Sci..

[36] Zhou X., Wang M., Zhang L., Liu Z., Su C., Wu M., Wei X., Jiang L., Hou J., Jiang Z. (2022). Hydroxypropyl Methylcellulose (HPMC) Reduces the Hardening of Fructose-Containing and Maltitol-Containing High-Protein Nutrition Bars during Storage. LWT.

[37] Zhang Z., Shi Y., Zheng H., Zhou Z., Wu Z., Shen D., Wang Y., Zhang Y., Wang Z., Fu B. (2021). A Hydroxypropyl Methylcellulose Film Loaded with AFCP Nanoparticles for Inhibiting Formation of Enamel White Spot Lesions. Int. J. Nanomed..

[38] Gong Y., Chen X., Wu W. (2024). Application of Fourier Transform Infrared (FTIR) Spectroscopy in Sample Preparation: Material Characterization and Mechanism Investigation. Adv. Sample Prep..

[39] Bashir S., Zafar N., Lebaz N., Mahmood A., Elaissari A. (2020). Hydroxypropyl Methylcellulose-Based Hydrogel Copolymeric for Controlled Delivery of Galantamine Hydrobromide in Dementia. Processes.

[40] Soe M.T., Chitropas P., Pongjanyakul T., Limpongsa E., Jaipakdee N. (2020). Thai Glutinous Rice Starch Modified by Ball Milling and Its Application as a Mucoadhesive Polymer. Carbohydr. Polym..

[41] Hong T., Yin J.-Y., Nie S.-P., Xie M.-Y. (2021). Applications of Infrared Spectroscopy in Polysaccharide Structural Analysis: Progress, Challenge and Perspective. Food Chem. X.

[42] Chang Y., Hu C., Yang R., He D., Wang X., Ning B., Sun H., Xiong Y., Tu J., Sun C. (2020). A Raman Imaging-Based Technique to Assess HPMC Substituent Contents and Their Effects on the Drug Release of Commercial Extended-Release Tablets. Carbohydr. Polym..

[43] Cherniienko A., Lesyk R., Zaprutko L., Pawełczyk A. (2024). IR-EcoSpectra: Exploring Sustainable Ex Situ and in Situ FTIR Applications for Green Chemical and Pharmaceutical Analysis. J. Pharm. Anal..

[44] Bumbrah G.S., Sharma R.M. (2016). Raman Spectroscopy—Basic Principle, Instrumentation and Selected Applications for the Characterization of Drugs of Abuse. Egypt. J. Forensic Sci..

[45] Rodríguez I., Gautam R., Tinoco A.D. (2020). Using X-Ray Diffraction Techniques for Biomimetic Drug Development, Formulation, and Polymorphic Characterization. Biomimetics.

[46] Holder C.F., Schaak R.E. (2019). Tutorial on Powder X-Ray Diffraction for Characterizing Nanoscale Materials. ACS Nano.

[47] von Schantz L., Schagerlöf H., Nordberg Karlsson E., Ohlin M. (2014). Characterization of the Substitution Pattern of Cellulose Derivatives Using Carbohydrate-Binding Modules. BMC Biotechnol..

[48] Gill P., Moghadam T.T., Ranjbar B. Differential Scanning Calorimetry Techniques: Applications in Biology and Nanoscience. https://pmc.ncbi.nlm.nih.gov/articles/PMC2977967/.

[49] Elgharbawy A.S., El Demerdash A.-G.M., Sadik W.A., Kasaby M.A., Lotfy A.H., Osman A.I. (2024). Enhancing the Biodegradability, Water Solubility, and Thermal Properties of Polyvinyl Alcohol through Natural Polymer Blending: An Approach toward Sustainable Polymer Applications. Polymers.

[50] Katona J., Njaradi S., Sovilj V., Petrovic L., Marceta B., Milanovic J. (2014). Rheological Properties of Hydroxypropylmethyl Cellulose/Sodium Dodecylsulfate Mixtures. J. Serbian Chem. Soc..

[51] Yoo Y.J., Um I.C. (2013). Examination of Thermo-Gelation Behavior of HPMC and HEMC Aqueous Solutions Using Rheology. Korea-Aust. Rheol. J..

[52] Reinoso D., Martín-Alfonso M.J., Luckham P.F., Martínez-Boza F.J. (2019). Rheological Characterisation of Xanthan Gum in Brine Solutions at High Temperature. Carbohydr. Polym..

[53] Zhang Q., Li X., Jasti B.R. (2021). Role of Physicochemical Properties of Some Grades of Hydroxypropyl Methylcellulose on in Vitro Mucoadhesion. Int. J. Pharm..

[54] HPMCTop Hypromellose (HPMC): Excipient Uses, Suppliers and Specifications. https://www.hpmctop.com/resources/hypromellose-excipient-uses-suppliers-and-specifications.html.

[55] Iglesias N., Galbis E., Romero-Azogil L., Benito E., Lucas R., García-Martín M.G., de-Paz M.-V. (2020). In-Depth Study into Polymeric Materials in Low-Density Gastroretentive Formulations. Pharmaceutics.

[56] Ghori M.U., Ginting G., Smith A.M., Conway B.R. (2014). Simultaneous Quantification of Drug Release and Erosion from Hypromellose Hydrophilic Matrices. Int. J. Pharm..

[57] Viridén A., Larsson A., Wittgren B. (2010). The Effect of Substitution Pattern of HPMC on Polymer Release from Matrix Tablets. Int. J. Pharm..

[58] Jiang Z., Ngai T. (2022). Recent Advances in Chemically Modified Cellulose and Its Derivatives for Food Packaging Applications: A Review. Polymers.

[59] Sarkar J., Mollick M.M.R., Chattopadhyay D., Acharya K. (2017). An Eco-Friendly Route of γ-Fe_2_O_3_ Nanoparticles Formation and Investigation of the Mechanical Properties of the HPMC-γ-Fe_2_O_3_ Nanocomposites. Bioprocess Biosyst. Eng..

[60] Hivechi A., Bahrami S.H. (2016). A New Cellulose Purification Approach for Higher Degree of Polymerization: Modeling, Optimization and Characterization. Carbohydr. Polym..

[61] Taokaew S. (2024). Bacterial Nanocellulose Produced by Cost-Effective and Sustainable Methods and Its Applications: A Review. Fermentation.

[62] Paramitasari D., Amelia O., Pudjianto K., Musa M., Rustiaty B., Supriyanti A., Meidiawati D.P., Putra O.N., Pramana Y.S., Yassaroh Y. (2025). Hydroxypropyl Cellulose Research over Two Decades (2005–2024): A Systematic Review with Bibliometric Analysis and Translational Insights. Polysaccharides.

[63] Isikgor F.H., Becer C.R. (2015). Lignocellulosic Biomass: A Sustainable Platform for the Production of Bio-Based Chemicals and Polymers. Polym. Chem..

[64] Hamed O., Al-Kerm R., Al-Kerm R., Qrareya H., Deghles A., Dagdag O. (2021). Carboxymethylated Pulp as Starting Point to Prepare Hydroxypropylmethyl Cellulose with Enhanced Gel Rheological Properties in an Aqueous Medium. Bioresources.

[65] Li W., Mortha G., Otsuka I., Ogawa Y., Nishiyama Y. (2023). Comparative Analysis of Molecular Weight Determination Techniques for Cellulose Oligomers. Cellulose.

[66] Fox S.C., Li B., Xu D., Edgar K.J. (2011). Regioselective Esterification and Etherification of Cellulose: A Review. Biomacromolecules.

[67] Klemm D., Philipp B., Heinze T., Heinze U., Wagenknecht W. (1998). Comprehensive Cellulose Chemistry.

[68] Hu H., You J., Gan W., Zhou J., Zhang L. (2015). Synthesis of Allyl Cellulose in NaOH/Urea Aqueous Solutions and Its Thiol–Ene Click Reactions. Polym. Chem..

[69] Adali M.B., Barresi A.A., Boccardo G., Pisano R. (2020). Spray Freeze-Drying as a Solution to Continuous Manufacturing of Pharmaceutical Products in Bulk. Processes.

[70] Li J., Zhao L., Lin X., Shen L., Feng Y. (2017). Co-Spray Drying with HPMC as a Platform to Improve Direct Compaction Properties of Various Tablet Fillers. AAPS PharmSciTech.

[71] Singh D. (2025). Advancements in Hydroxypropyl Methylcellulose–Polyethylene Glycol Hybrid Excipients and Their Nanoformulations for Controlled and Ligand-Directed Biologic Delivery. Naunyn-Schmiedeberg’s Arch. Pharmacol..

[72] Martínez L.M., Cruz-Angeles J., Vázquez-Dávila M., Martínez E., Cabada P., Navarrete-Bernal C., Cortez F. (2022). Mechanical Activation by Ball Milling as a Strategy to Prepare Highly Soluble Pharmaceutical Formulations in the Form of Co-Amorphous, Co-Crystals, or Polymorphs. Pharmaceutics.

[73] Virden A. Method Development for Laser-Diffraction Particle-Size Analysis. https://www.pharmtech.com/view/method-development-laser-diffraction-particle-size-analysis.

[74] Moussa E., Siepmann F., Flament M.P., Benzine Y., Penz F., Siepmann J., Karrout Y. (2019). Controlled Release Tablets Based on HPMC:Lactose Blends. J. Drug Deliv. Sci. Technol..

[75] Rana R.H., Rana M.S., Tasnim S., Haque M.R., Kabir S., Amran M.S., Chowdhury A.A. (2022). Characterization and Tableting Properties of Microcrystalline Cellulose Derived from Waste Paper via Hydrothermal Method. J. Appl. Pharm. Sci..

[76] Pani N.R., Nath L.K. (2014). Development of Controlled Release Tablet by Optimizing HPMC: Consideration of Theoretical Release and RSM. Carbohydr. Polym..

[77] Pan P., Svirskis D., Waterhouse G.I.N., Wu Z. (2023). Hydroxypropyl Methylcellulose Bioadhesive Hydrogels for Topical Application and Sustained Drug Release: The Effect of Polyvinylpyrrolidone on the Physicomechanical Properties of Hydrogel. Pharmaceutics.

[78] Galata D.L., Könyves Z., Nagy B., Novák M., Mészáros L.A., Szabó E., Farkas A., Marosi G., Nagy Z.K. (2021). Real-Time Release Testing of Dissolution Based on Surrogate Models Developed by Machine Learning Algorithms Using NIR Spectra, Compression Force and Particle Size Distribution as Input Data. Int. J. Pharm..

[79] Greiderer A., Steeneken L., Aalbers T., Vivó-Truyols G., Schoenmakers P. (2011). Characterization of Hydroxypropylmethylcellulose (HPMC) Using Comprehensive Two-Dimensional Liquid Chromatography. J. Chromatogr. A.

[80] Boonen J., Veryser L., Taevernier L., Roche N., Peremans K., Burvenich C., De Spiegeleer B. (2014). Risk Evaluation of Impurities in Topical Excipients: The Acetol Case. J. Pharm. Anal..

[81] Amarji B., Kulkarni A., Deb P.K., Deepika D., Maheshwari R., Tekade R.K. (2018). Package Development of Pharmaceutical Products. Dosage Form Design Parameters.

[82] Guo J., Dong S., Ye M., Wu X., Lv X., Xu H., Li M. (2022). Effects of Hydroxypropyl Methylcellulose on Physicochemical Properties and Microstructure of κ-Carrageenan Film. Foods.

[83] Zhang Y., Jiang Z., Zhu Y., Zhang J., Ren Q., Huang T. (2021). Effects of Redispersible Polymer Powders on the Structural Build-up of 3D Printing Cement Paste with and without Hydroxypropyl Methylcellulose. Constr. Build. Mater..

[84] Gu X., Li X., Zhang W., Gao Y., Kong Y., Liu J., Zhang X. (2021). Effects of HPMC on Workability and Mechanical Properties of Concrete Using Iron Tailings as Aggregates. Materials.

[85] Batista I.L.R., Cabral K.C., de Souza W.R.M., de Sousa Fontes A.É.M., Martinelli A.E. (2024). Influence of Hydroxypropylmethylcellulose (HPMC) on Thermal and Mechanical Performance of Cementitious Rendering Mortars. Mater. Struct..

[86] Milenkova S., Marudova M., Viraneva A. (2026). Biodegradable Polymer Films Based on Hydroxypropyl Methylcellulose and Blends with Zein and Investigation of their Potential as Active Packaging Material. Coatings.

[87] López de Dicastillo C., Velásquez E., Rojas A., Garrido L., Moreno M.C., Guarda A., Galotto M.J. (2023). Developing Core/Shell Capsules Based on Hydroxypropyl Methylcellulose and Gelatin through Electrodynamic Atomization for Betalain Encapsulation. Polymers.

[88] Dave S., Soni V., Shah S.A., Kraft W.K., Kaushal G. (2026). Hydroxypropyl Methylcellulose as a Mucoadhesive Polymer in Ethanol-Free Buprenorphine Gel for Neonatal Sublingual Delivery. Polymers.

[89] Kraisit P., Hirun N., Limpamanoch P., Sawaengsuk Y., Janchoochai N., Manasaksirikul O., Limmatvapirat S. (2024). Effect of Cremophor RH40, Hydroxypropyl Methylcellulose, and Mixing Speed on Physicochemical Properties of Films Containing Nanostructured Lipid Carriers Loaded with Furosemide Using the Box–Behnken Design. Polymers.

[90] Khiste R., Bhapkar N., Kulkarni N. (2021). A Review on Applications of Hydroxy Propyl Methyl Cellulose and Natural Polymers for the Development of Modified Release Drug Delivery Systems. Res. J. Pharm. Technol..

[91] Ozon E.A., Burloiu A.M., Musuc A.M., Manda G., Anuta V., Dinu-Pîrvu C.E., Lupuliasa D., Neagoe I.V., Anastasescu M., Socoteanu R.P. (2025). Cellulose-Derived Gels for Topical Delivery: HPMC as a Functional Matrix for Porphyrinic Photosensitizers. Gels.

[92] Kadry H., Al-Hilal T.A., Keshavarz A., Alam F., Xu C., Joy A., Ahsan F. (2018). Multi-Purposable Filaments of HPMC for 3D Printing of Medications with Tailored Drug Release and Timed-Absorption. Int. J. Pharm..

[93] Hardy I.J., Cook W.G., Melia C.D. (2006). Compression and Compaction Properties of Plasticised High Molecular Weight Hydroxypropylmethylcellulose (HPMC) as a Hydrophilic Matrix Carrier. Int. J. Pharm..

[94] Phaechamud T., Mesnukul A., Yodkhum K. (2009). Solid Dispersion Matrix Tablet Comprising Indomethacin-PEG-HPMC Fabricated with Fusion and Mold Technique. Indian J. Pharm. Sci..

[95] Andonova V., Zagorchev P., Katsarov P., Kassarova M. (2015). Eye Drops with Nanoparticles as Drug Delivery Systems. Int. J. Pharm. Pharm. Sci..

[96] Akbal Dağistan Ö., Arslan M. (2024). Selection of the Polymers Used in Oral Dispersible Films via Analytical Hierarchy Process. J. Res. Pharm..

[97] Wang X., Sun H. (2025). Synergistic Effect of Microbial-Induced Carbonate Precipitation Modified with Hydroxypropyl Methylcellulose on Improving Loess Disintegration and Seepage Resistance. Polymers.

[98] Lim C., Song Y.H., Song Y., Seo J.H., Hwang D.S., Lee D.W. (2021). Adaptive Amphiphilic Interaction Mechanism of Hydroxypropyl Methylcellulose in Water. Appl. Surf. Sci..

[99] Polamaplly P., Cheng Y., Shi X., Manikandan K., Kremer G.E., Qin H. (2019). 3D Printing and Characterization of Hydroxypropyl Methylcellulose and Methylcellulose for Biodegradable Support Structures. Procedia Manuf..

[100] Zhu W., Yuan M., He F., Zhao Y., Xiao Z., Wang Q., Meng F., Tang Q. (2022). Effects of Hydroxypropyl Methylcellulose (HPMC) on the Reinforcement of Sand by Microbial-Induced Calcium Carbonate Precipitation (MICP). Appl. Sci..

[101] Wang S., Liu C., Chen H., Zhu A., Qian F. (2020). Impact of Surfactants on Polymer Maintained Nifedipine Supersaturation in Aqueous Solution. Pharm. Res..

[102] Bading M., Olsson O., Kümmerer K. (2024). Analysis of Environmental Biodegradability of Cellulose-Based Pharmaceutical Excipients in Aqueous Media. Chemosphere.

[103] Hart-Cooper W.M., Kalla N., Klamczynski A., Torres L., Glenn G.M., Cunniffe J., Johnson K., Orts W.J. (2024). Predicting Environmental Biodegradability Using Initial Rates: Mineralization of Cellulose, Guar and Their Semisynthetic Derivatives in Wastewater and Soil. Front. Mater..

[104] The Dow Chemical Company Environment, Health and Safety (EH&S) Policy. https://corporate.dow.com/en-us/about-dow/company/public-policy/environment-health-safety.html.

[105] U.S. Environmental Protection Agency (EPA) Cellulose Products Manufacturing: National Emission Standards for Hazardous Air Pollutants (NESHAP). https://www.epa.gov/stationary-sources-air-pollution/cellulose-products-manufacturing-national-emission-standards.

[106] Kamide K. (2005). Characterization of Molecular Structure of Cellulose Derivatives. Cellulose and Cellulose Derivatives.

[107] Jin C., Wu F., Hong Y., Shen L., Lin X., Zhao L., Feng Y. (2023). Updates on Applications of Low-Viscosity Grade Hydroxypropyl Methylcellulose in Coprocessing for Improvement of Physical Properties of Pharmaceutical Powders. Carbohydr. Polym..

