# Supplementary material for: Exploring the Versatility and Sustainability of Hydroxypropyl Methylcellulose (HPMC) in Modern Chemical Industry
Source: Polymers (Basel). 2026 Apr 30;18(9):1105. doi: 10.3390/polym18091105 (PMC13165829; doi:10.3390/polym18091105)
Supplement: Supplementary file 1 [file polymers-18-01105-s001.zip › polymers-4266883-supplementary.pdf]

Table S1: Analytical techniques for the characterization of HPMC together with the information provided by each technique and examples of practical insights (summary and compilation on the basis of information and references cited within Section 2).

Technique	Information on HPMC	Examples of Practical Insights
**Scanning Electron Microscopy (SEM)**<br>Focused electron beam for high-resolution surface imaging (morphology/ topography).	Surface structure, morphology, particle size of HPMC films/ powders; film uniformity and porosity.	Evaluating HPMC film coatings in tablets; assessing surface changes after processing or drug incorporation.
**Transmission Electron Microscopy (TEM)**<br>Electron transmission through ultrathin samples for nanoscale internal imaging.	Internal microstructure; dispersion of additives/fillers within HPMC matrices.	Analyzing HPMC nanocomposites; studying filler distribution and polymer–filler interactions.
**FTIR (Fourier Transform Infrared Spectroscopy)**<br>Infrared absorption to identify chemical bonds/ functional groups.	Chemical structure; functional groups; interactions (e.g., H-bonding, drug–polymer interactions).	Confirming chemical modifications; drug compatibility studies; monitoring chemical stability during storage.
**Raman Spectroscopy**<br>Inelastic scattering of monochromatic light for vibrational information.	Molecular structure and crystallinity; detection of chemical interactions (complementary to FTIR).	Tracking polymer crystallinity; monitoring drug dispersion/ interactions in HPMC formulations.
**X-ray Diffraction (XRD)**<br>X-ray diffraction patterns to determine crystalline structure.	Crystallinity vs. amorphous content; changes due to substitution or blending.	Evaluating crystallinity changes in films; studying drug–polymer miscibility and solid-state forms.
**NMR (Nuclear Magnetic Resonance)**<br>Magnetic fields/radio waves for molecular structure and dynamics.	Substitution patterns; chemical structure; (where applicable) molecular weight distribution.	Confirming degree/ type of substitution; studying chain dynamics; supporting structural elucidation.
**Differential Scanning Calorimetry (DSC)**<br>Heat flow associated with thermal transitions.	Tg; degradation onset; moisture-related effects; compatibility assessment.	Optimizing processing; detecting amorphous dispersions; designing blends with tailored thermal properties.
**Thermogravimetric Analysis (TGA)**<br>Mass change vs. temperature for thermal stability/composition.	Decomposition profile; thermal stability; indications of interactions/volatiles (e.g., moisture).	QC; optimizing thermal robustness; comparing grades/ formulations for stability.
**Rheology**<br>Deformation/ flow measurements (viscosity, viscoelasticity).	Flow behavior; gelation behavior; polymer interactions in solution; sensitivity to MW/ substitution.	Tuning release profiles; controlling gelation; assessing dispersion in composites; tailoring solution properties.
